# Multi‐Omics Data Reveal Estrogen‐Driven Dysregulation and Stromal‐Epithelial Signaling Alterations in Endometrial Polyps

**DOI:** 10.1096/fj.202504234R

**Published:** 2026-03-05

**Authors:** Tingwei Chen, Bo Zhang, Zhengli Zhou, Naixue Yang, Ting Liu, Huimei Zhang, Yu Yin, Xiaomei Wu, Xiaozhuo Li, Tao Yu, Xiaodie Wang, Tianqing Li, E. Dong

**Affiliations:** ^1^ State Key Laboratory of Primate Biomedical Research; Institute of Primate Translational Medicine Kunming University of Science and Technology Kunming China; ^2^ Yunnan Key Laboratory of Primate Biomedical Research Kunming China; ^3^ Southwest United Graduate School Kunming China; ^4^ The First People’ Hospital of Yunnan Province The Affiliated Hospital of Kunming University of Science and Technology Kunming China

**Keywords:** endometrial polyps, estrogen signaling, multi‐omics, organoid, single‐cell RNA sequencing, stromal‐epithelial crosstalk, transcriptomics

## Abstract

Endometrial polyps (EPs) are common uterine lesions associated with abnormal uterine bleeding and infertility, yet their pathogenesis remains poorly defined. Here, we performed single‐cell RNA sequencing of normal endometrium, para‐polyp, and polyp tissues, identifying distinct cellular compositions and transcriptional programs. EPs showed enhanced estrogen signaling and increased epithelial proliferation, accompanied by decreased expression of cytokines and reduced T cell cytotoxicity. Notably, we observed epithelial subpopulations with elevated copy number variations and transcription factors associated with hyperplasia. Cell–cell communication analyzes revealed aberrant stromal‐epithelial crosstalk, characterized by upregulated WNT, IGF, and VEGF signaling originating from stromal cells. Spatial transcriptomic analyzes further demonstrated enhanced WNT signaling between stromal and epithelial compartments in endometrial cancer. In vitro glandular organoid models showed that epithelial transcriptional alterations contribute to polyp formation. These findings highlight a critical role of stromal‐epithelial interactions in EP development and suggest potential therapeutic targets.

## Graphical abstract

Single‐cell transcriptomic profiling reveals that endometrial polyps arise from estrogen‐driven disruption of stromal‐epithelial signaling. Stromal cells exhibit enhanced WNT, IGF, and VEGF pathways, promoting epithelial proliferation and vascular remodeling. These changes are accompanied by increased inflammatory cytokine expression and impaired T cell cytotoxicity, collectively establishing a permissive microenvironment for polyp formation and growth, and highlighting stromal–epithelial interactions as central regulators of endometrial pathology.

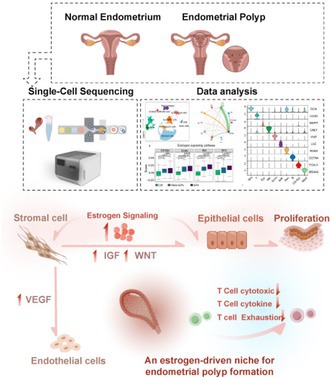

## 1 Introduction

Endometrial polyps (EPs) are overgrowths of endometrial glands and stroma within the uterine cavity and are classified into hyperplastic, atrophic, functional, and adenomyomatous types [1, 2]. They are a common gynecological condition in women of reproductive age, with a prevalence of approximately 25% [3]. EPs can present as single or multiple lesions that fill the uterine cavity and are categorized as sessile, pedunculated, or prolapsing through the cervix [2, 3]. Clinical symptoms include abnormal uterine bleeding, abdominal or pelvic pain, and infertility [4]. While approximately 23% of incidental polyps may resolve spontaneously, those larger than 2 cm are unlikely to regress [5]. Large polyps can act as mechanical barriers to embryo implantation or release factors detrimental to implantation [6]. Hysteroscopic polypectomy is the primary treatment and has been shown to improve pregnancy outcomes compared to non‐intervention [7]. Although most EPs are benign, 0%–13% may undergo malignant transformation, with the risk increasing with patient age and menopausal status [2, 8, 9, 10, 11].

Despite their prevalence, the etiology of EPs remains poorly understood. EPs are associated with endometrial hyperplasia, and unopposed estrogen is considered a major risk factor [12]. Other proposed mechanisms include monoclonal endometrial overgrowth, aromatase overexpression [13], genetic mutations [14], dysregulation of high‐mobility group transcription factors, resistance to apoptosis [15], and genetic predisposition [16]. However, comprehensive single‐cell sequencing studies examining gene expression in EPs and adjacent tissues are lacking.

The advent of single‐cell transcriptomic technologies has revolutionized our understanding of the human endometrium, providing unprecedented resolution into cellular composition, lineage‐specific gene expression patterns, and regulatory pathways underlying endometrial transformation across the human menstrual cycle [17]. These technologies have also been instrumental in uncovering aberrant cellular states and molecular disruptions associated with various pathological conditions, such as endometriosis [18], thin endometrium [19], and Asherman's syndrome [20, 21]. However, comprehensive single‐cell analysis focused specifically on EPs remains limited. In‐depth characterization of EPs and their adjacent tissues at the single‐cell level offers a powerful approach to decipher cell‐specific transcriptomic alterations and dynamic changes in tissue architecture. Such comparative analyzes are essential for uncovering the mechanisms driving polyp development and their potential links to local microenvironmental disturbances. A recent single‐cell sequencing study revealed that aberrant expression of WT1 in mast cells may promote the formation of endometrial polyps [22]. Nonetheless, cellular interactions within the endometrial microenvironment remain critical to understanding EP pathogenesis.

Chronic endometritis (CE) is frequently linked to the development of EPs [23]. Moreover, EPs are often accompanied by excessive glandular proliferation [2, 16], which may result from estrogen‐mediated hyperactivation of epithelial cells [24, 25]. However, the precise mechanisms driving this hyperplasia remain unclear. Thus, elucidating both the extrinsic signals and intrinsic factors that promote glandular proliferation is critical to understanding the formation and progression of EPs.

Given these insights, elucidating the transcriptional regulation and cellular interactions within endometrial polyps is pivotal for understanding their pathophysiology. In this study, we constructed a single‐cell transcriptomic atlas of EPs, para‐EPs, and normal endometrial tissues. Our analysis revealed substantial alterations in cellular composition, gene expression, and intercellular communication associated with EP development. We identified enhanced estrogen signaling and epithelial proliferation in polyp epithelium, accompanied by low‐cytotoxic T cell signatures and reduced expression of cytokines. Notably, we detected genomic instability and transcriptional heterogeneity within epithelial subpopulations, implicating CNV‐driven gene regulation in EP progression. Furthermore, we uncovered aberrant WNT‐mediated stromal‐epithelial interactions and rewired stromal‐centered signaling networks involving VEGF, IGF, and LIF pathways in EPs. Spatial transcriptomic analysis further revealed that upregulation of WNT signaling between stromal and epithelial cells may contribute to endometrial cancer development. Transcriptomic profiling of ex vivo glandular organoids indicated that epithelial cells in polyps, regardless of estrogen exposure, exert pro‐inflammatory effects. These findings highlight the critical role of epithelial‐stromal‐immune crosstalk in driving glandular overgrowth and inflammation, offering novel insights into the pathogenesis of EPs and potential therapeutic targets for associated reproductive complications.

## 2 Methods

### 2.1 Ethics and Clinical Sample Collection

This study complied with all relevant ethical regulations and was approved by the Ethics Committee of First People's Hospital of Yunnan Province, affiliated with Kunming University of Science and Technology (ethics number: KHLL2021‐KY049) and has been performed in accordance with the principles of the Declaration of Helsinki. All patients were fully informed about the use of their samples in this study and provided written informed consent. The information of all endometrial samples is shown in Table S1.

### 2.2 Endometrium Tissue Dissociation and Preparation of Single‐Cell Suspensions

Freshly isolated endometrial tissues were immediately placed in ice‐cold DPBS (Gibco, 14190144) containing 1% fetal bovine serum (FBS, Gibco 10 099–141) and transported on ice to maintain cell viability. A two‐step dissociation protocol was used to dissociate endometrium tissue and separate the tissues into stromal fibroblast‐ and epithelium‐enriched single‐cell suspensions. Tissues were washed 2–3 times with PBS, dissected in a 3.5 cm Petri dish to remove blood and mucus, and cut into smaller pieces. The samples were then transferred to 10 mL digestion medium containing 1 mg/mL Collagenase Type IV (Gibco, 17104‐019), 2.5 IU/mL Dispase (Corning, 354235), and 100 μg/mL DNase I (Sigma‐Aldrich, 11 28493 001) in DMEM medium. Enzymatic digestion was carried out at 37°C with shaking at 50 r.p.m. for 20–30 min. This primary enzymatic step dissociates stromal fibroblasts into single cells while leaving epithelial glands and lumen largely intact.

Cell suspensions was filtered through a 40‐μm nylon cell strainer (BD Falcon, 352340) and stromal cells passing through epithelial cell retained on the strainer. The epithelial cells were then backwashed with DMEM and dissociated further by incubating in 400 μL of TrypLE Select (Life technology) for 20 min at 37°C, with intermittent pipetting to facilitate homogenization. Stromal cell suspensions were treated with RBC lysis buffer (Invitrogen, 00–4333‐57) to remove red blood cells. The entire cell suspension was filtered again through a 40‐μm cell strainer and centrifuged at 1000 r.p.m. for 5 min. Dissociated cells were washed with PBS containing 0.04% Bovine Serum Albumin (BSA; Sigma‐Aldrich, B2064), centrifuged at 500 g for 5 min, and the resulting cell pellet was collected. Cell viability was determined by Trypan blue (Invitrogen, T10282) staining and then cells were suspended in PBS with 0.04% BSA at a density of about 1 × 10^6^ cells/mL. Cells were then kept on ice for single‐cell sequencing.

### 2.3 Chromium 10× Single‐Cell Library Construction and Sequencing

Single‐cell suspensions were converted to barcoded scRNA‐seq libraries according to standard protocols of the Chromium single‐cell 3′ kit (V3 chemistry). GEM generation and barcoding, reverse transcription, cDNA generation and library construction were performed according to the standard manufacturer's protocol. The libraries were applied to pair‐end sequencing with read lengths of 150 nt on Illumina HiSeq Xten platform (the Illumina NovaSeq6000 platform for PE150 sequencing).

### 2.4 Filtering Low‐1uality Cells and Eliminating Doublet

Cell Ranger (version 5.0.1) was used to map reads to the GRCh38 human genome and acquire gene counts to generate expression matrix files. scRNA‐seq data processing and analysis were implemented using the Seurat v4.3.0.1 R package. All functions were executed with default parameters unless otherwise stated. Then data were integrated to eliminate batch effects across samples using the Seurat functions FindIntegrationAnchors and IntegrateData. After integration, the objects were normalized and scaled using the NormalizeData and ScaleData functions. Low‐quality cells defined as those with fewer than 200 or more than 7500 detected genes, or with over 20% mitochondrial gene expression were excluded from the analysis. Following quality control, doublets were identified using the DoubletFinder method. The normalized gene expression matrix generated after data preprocessing was used to identify the major cell clusters by applying dimension reduction and clustering.

### 2.5 Dimension Reduction and Identification of Major Cell Clusters

Principal component analysis (PCA) dimensionality reduction was run using default parameters. The top 30 principal components were adopted to identify distinct clusters of cells using the graph‐based clustering method with the FindClusters function (resolution = 0.6). The cell clusters were visualized using uniform manifold approximation and projection (UMAP). We calculated the marker genes using the FindAllMarkers function with the Wilcoxon rank sum test algorithm under the following criteria: (1) ∣log2FC∣ > 0.25; (2) *p* value < 0.05; and (3) min.pct > 0.25. The cell type annotation of each cluster was done based on the expression from DEG and canonical markers in the literature with Feature plot and violin plots. A heatmap was generated using pheatmap (version 1.0.12) to visualize the top three marker genes for each annotated cell type.

### 2.6 Cell Cycle Analysis

Cell cycle phase assignment was performed using the CellCycleScoring() function in the Seurat R package (v4.3.0), which classifies individual cells into G1, S, or G2/M phases based on canonical cell cycle gene expression. The distribution of cell cycle states across different experimental groups was visualized using dimensionality reduction via the DimPlot() function. Quantification of phase proportions was presented using bar plots, and statistical comparisons between groups were performed using the Wilcoxon rank‐sum test.

### 2.7 Compositional Analysis of Cell Types and T Cell Subsets Across Groups

The relative abundance of each major cell type and T cell subset was quantified by calculating their proportions within individual samples. These compositional profiles were visualized using grouped and stacked bar plots generated with the ggplot2 package (v3.5.1). Statistical comparisons between experimental groups were conducted using the *t*‐test, and significant *p*‐values were directly annotated on the corresponding plots.

### 2.8 Differential Gene Expression Analysis

Differential gene expression analysis was conducted using the FindMarkers() function in Seurat (v4.3.0) based on the Wilcoxon rank‐sum test. To identify cell type‐specific marker genes, the FindAllMarkers() function was applied. Genes with an average log_2_ fold change (|avg_log2FC|) > 0.25 and an adjusted *p*‐value < 0.05 were considered differentially expressed genes (DEGs) or markers. Functional enrichment analyzes of Gene Ontology (GO) terms and Kyoto Encyclopedia of Genes and Genomes (KEGG) pathways were performed using the clusterProfiler R package (v3.18.1). Significantly enriched pathways or terms were visualized as dot plots using ggplot2 (v3.5.1).

### 2.9 Signature Scores

Pathway‐associated gene sets (e.g., cytotoxicity, cytokine signaling, exhaustion, estrogen signaling) were obtained from the KEGG and GO databases using the msigdbr R package (v7.5.1). Signature scores were computed using Seurat's AddModuleScore() function (v4.3.0), yielding per‐cell pathway activity scores across all annotated cell types. Group‐wise comparisons were visualized using box plots and stacked violin plots generated with ggplot2 (v3.5.1). Statistical significance was assessed using Wilcoxon rank‐sum tests for pairwise comparisons within each cell type, with *p*‐values directly annotated on the plots.

### 2.10 Single‐Cell RNA‐Seq Analysis Inferring Copy Number Variation

Copy number variation analysis of epithelial subpopulations from Ctrl, Para‐EPs, and EPs groups was performed using the inferCNV R package (v1.18.1), with stromal cells from control samples serving as the reference. The standard pipeline was used to infer CNV profiles, resulting in the classification of epithelial cells into five distinct CNV groups. CNV patterns were visualized using: (i) annotated heatmaps generated with ComplexHeatmap (v2.20.0), incorporating sample group annotations, and (ii) box plots generated with ggplot2 (v3.5.1) to compare CNV scores across cell types and CNV clusters.

### 2.11 Cell Chat Analysis

To investigate cell–cell communication, we performed CellChat analysis (https://github.com/sqjin/CellChat) separately for the Ctrl, Para‐EPs, and EPs groups. Intercellular signaling networks were inferred for each group, followed by joint manifold learning and functional classification of signaling pathways. Information flow (i.e., communication probability) between all pairs of cell types was quantified and compared across groups. Visualization strategies included: Line plots (generated using ggplot2 v3.5.1) illustrating global differences in directional communication strength; Heatmaps (via netVisual_heatmap) depicting pairwise communication probability differences; Stacked bar plots and dot plots (via rankNet) showing pathway‐specific communication strength; Chord diagrams (via netVisual_aggregate using the circlize package) to visualize pathway‐level interactions between cell types; Violin plots to compare WNT ligand expression across groups; Bubble plots to highlight differences in WNT ligand‐receptor pair interactions.

### 2.12 SCENIC Analysis

Transcription factor (TF) regulon analysis across epithelial CNV groups was performed using pySCENIC (v0.12.1), following the standard three‐step regulatory inference workflow. First, gene co‐expression networks were constructed using the GRNBoost2 algorithm on group‐specific expression matrices. Next, regulons were refined via cisTarget by integrating motif enrichment analysis based on hg38 position weight matrices, thereby identifying direct TF targets. Finally, AUCell scoring was applied to quantify regulon activity at the single‐cell level. The resulting normalized AUC matrices were visualized as hierarchically clustered heatmaps using ComplexHeatmap (v2.20.0), with column annotations denoting CNV group identity and row annotations indicating regulon specificity scores, thereby highlighting distinct TF activity patterns across CNV‐defined epithelial subpopulations.

### 2.13 Location of Cell Types in Visium Data

A reference cell signature matrix was constructed from the processed single‐cell RNA sequencing data. Genes exhibiting high expression levels in the ambient RNA signal were identified and excluded from both the reference and the spatial transcriptomics object to enhance mapping specificity. Following the established workflow detailed in the cell2location documentation, the cell2location model (v0.1.4) was trained to estimate the abundance of cell subpopulations across spatial locations. All computations were performed in a Python (v3.12) environment using the software's default parameters.

### 2.14 Establishment of Endometrial Organoids

The epithelial fraction collected from the back‐flush of the 40 μm cell strainer was resuspended in 70% Matrigel() and plated as 25 μL domes in 48‐well plates. Each well was overlaid with 250 μL of gland culture medium, which was composed of the following components: advanced DMEM/F12 medium (Life Technologies,12 634 010), N2 supplement (Life Technologies, 17502048, 100×), B27 supplement minus vitamin A (Life Technologies,12587010, 50×), glutaMAX Supplement (Gibco, 35050061, 2 mM), N‐Acetyl‐L‐cysteine (Sigma, A9165‐5G, 1.25 mM), Nicotinamide (Sigma, N0636, 1 mM), recombinant human NOGGIN (Peprotech, 120‐10C, 100 ng/mL), recombinant human Rspondin‐1 (BioTechne 4645‐RS, 70 ng/mL), recombinant human EGF (Peprotech, AF‐100‐15, 50 ng/mL), Recombinant human FGF‐10 (Peprotech, 100‐26, 100 ng/mL), Recombinant human HGF (Peprotech, 100‐39, 50 ng/mL), A83‐01 (System Biosciences, ZRD‐A8‐02, 500 nM), Y‐27632 (Merck, 688000, 10 μM). The organoids formed within 3–4 days and were passaged according to growth and confluency within the Matrigel. Organoid passaging and cryopreservation were performed as described by Margherita Y. Turco et al. [26].

### 2.15 Hormone Treatment of Endometrial Organoids

The hormone treatment protocol for inducing the proliferative phase of the endometrium was slightly modified from the method described by Thomas E. Spencer et al. [27], with organoids treated with 10 nM estradiol (E2, Sigma, E1024) or vehicle (100% ethanol) for 7 days.

### 2.16 Immunofluorescence Staining

Organoid culture medium was removed, and the organoids were fixed with 4% paraformaldehyde for 30 min. After washing with PBS, organoids were embedded in OCT compound and frozen at −40°C. Cryosections were cut at a thickness of 5 μm and air‐dried for 20 min at 37°C. Sections were again fixed in 4% paraformaldehyde for 5 min and washed with PBS. Sections were then permeabilized and blocked with 100–200 μL of 3% BSA containing 0.4% Triton X‐100 for 3–4 h at room temperature or overnight at 4°C. After washing three times with 0.05% Tween‐20 in PBS, sections were incubated overnight at 4°C with primary antibodies (Ki67 antibdy, 1:600, R&D system, AF7649‐SP; Progesterone Receptor A/B antibody, 1:500, Cell Signaling Technology, 8757S; Cytokeratin 7 antibody (ck7), 1:500, abcam, ab181598; CK7, 1:500, invitrogen, MA1‐06316). The following day, sections were incubated with Alexa Fluor 488/568/647‐conjugated secondary antibodies (1:500 dilution) and DAPI (1:1000 dilution) for 2 h at room temperature. Slides were then washed three times with PBS for 10 min each. Finally, 10–20 μL of 50% glycerol was added, and coverslips were mounted. Fluorescent images were captured using a Leica SP8 or Leica X confocal microscope.

### 2.17 Immunohistochemistry

Endometrium tissue samples were fixed in 4% paraformaldehyde and embedded in paraffin. After antigen retrieval to activate endogenous peroxidase, the sections were incubated in 3% hydrogen peroxide for 30 min. The samples were then blocked with 10% normal goat serum for 1 h. The expression of KI67 (AF7649‐SP, R&D system), ESR1 (13 258 s, Cell Signaling Technology), PGR (8757S, Cell Signaling Technology) were determined by incubating the sections with the primary antibody at 4°C overnight. The slides were subsequently incubated with the secondary antibody dilution and then treated with DAB solution for 1 h. Hematoxylin solution was briefly applied for 15 s to stain the nuclei. Finally, the slides were examined under a microscope.

### 2.18 Bulk RNA‐Sequencing

Total RNA was extracted from endometrial organoids. RNA quality control was performed by assessing purity using a spectrophotometer, quantifying concentration with a fluorometric assay, and evaluating integrity through microfluidic analysis. Sequencing libraries were constructed from high‐quality total RNA using a standard mRNA enrichment and strand‐specific protocol. Briefly, polyadenylated mRNA was purified using oligo (dT) magnetic beads and fragmented. First‐strand cDNA was synthesized with random hexamers, followed by second‐strand synthesis. The double‐stranded cDNA was purified, end‐repaired, adenylated, and ligated to sequencing adapters. The final cDNA library was generated after size selection and PCR amplification. Library quality was verified, and sequencing was performed on an Illumina NovaSeq 6000 platform in paired‐end 150 bp mode.

### 2.19 Gene Expression Analysis by Real‐Time PCR

Total RNA was extracted from proliferative‐phase human endometrial tissues using TRIzol reagent (Invitrogen). Reverse transcription was performed using an oligo (dT) primer (TaKaRa). The resulting cDNA was subjected to real‐time quantitative RT‐PCR analysis to assess the expression of target genes using SYBR Premix (Bio‐Rad) and appropriate primers specific for human genes. The fold change in the mRNA levels was determined via the 2^−ΔΔCt^ method. Primer sequences were as follows: IGF1 (forward, CTCTTCAGTTCGTGTGTGGAGAC; reverse, CAGCCTCCTTAGATCACAGCTC), VEGFA (forward, TTGCCTTGCTGCTCTACCTCCA; reverse, GATGGCAGTAGCTGCGCTGATA), and GAPDH (forward, GTCTCCTCTGACTTCAACAGCG; reverse, ACCACCCTGTTGCTGTAGCCAA).

### 2.20 Transcriptomic Analysis Using the HISAT2‐StringTie Workflow

RNA‐seq data processing and transcriptome assembly were performed using a standard workflow. Briefly, sequencing reads were aligned to the human reference genome GRCh38.p14 (Genome Reference Consortium Human Build 38) using HISAT2 (v2.2.1). The resulting alignments were converted to sorted BAM files using SAMtools (v1.20). Transcript assembly and quantification were subsequently performed with StringTie (v2.2.3) using the sorted BAM files as input and guided by the GENCODE v45 gene annotation. All steps were executed using the software's default parameters.

### 2.21 Principal Component Analysis (PCA)

Principal Component Analysis (PCA) was performed to assess the transcriptomic heterogeneity across samples. The analysis was conducted using the top 2000 most highly variable genes, selected based on their normalized dispersion across the dataset. The expression matrix of these selected genes was log‐transformed, centered to zero mean, and scaled to unit variance. Principal components were computed from the preprocessed data matrix using singular value decomposition. The results were visualized using ggplot2 (v3.5.0) in R (v4.4.0), where samples were represented as points in a two‐dimensional scatter plot based on their coordinates along the first two principal components.

### 2.22 Differential Gene Expression Analysis

Differential gene expression analysis was performed to identify genes with significant expression changes. The raw gene count matrix was analyzed using DESeq2 (version 1.42.1), which applies a negative binomial generalized linear model to assess statistical significance. Genes were defined as differentially expressed based on the following thresholds: adjusted *p*‐value < 0.05 and absolute log2 fold change ≥ 1. Results were visualized using ggplot2 (version 3.5.1) in R through a volcano plot, illustrating the relationship between the magnitude of expression change (log2 fold change) and statistical significance (−log10 adjusted *p*‐value). To emphasize genes with the most substantial changes, data points were labeled if they met either of the following criteria: absolute log2 fold change > 10 or −log10 adjusted *p*‐value > 8.

### 2.23 Functional Enrichment Analysis

Functional enrichment analysis was performed to interpret the biological significance of the identified gene sets. Predefined gene lists were analyzed using KOBAS (http://kobas.cbi.pku.edu.cn/) to identify significantly overrepresented Gene Ontology (GO) terms and Kyoto Encyclopedia of Genes and Genomes (KEGG) pathways. The enrichment results for these selected terms were visualized using ggplot2 (version 3.5.1) in R through a diverging bar plot arrangement. This visualization approach displays the enrichment significance of separate gene sets for each functional term on opposing sides of the axis, enabling effective comparative analysis.

### 2.24 Survival Analysis

Transcriptomic and corresponding clinical data for TCGA tumor samples were obtained from The Cancer Genome Atlas (TCGA) database. For each cancer type, cumulative signature scores for genes involved in each pathway were calculated and used as predictors in survival analyzes. Overall survival was defined as the time from initial diagnosis to death or last follow‐up. Univariable Cox proportional hazards regression models were fitted to evaluate the association between pathway‐specific cumulative signature scores and overall survival. All models were adjusted for patient age, sex, and tumor stage as covariates. Hazard ratios (HRs) and 95% confidence intervals (CIs) were estimated for each pathway across different cancer types. To account for multiple hypothesis testing, P values were adjusted using the Benjamini–Hochberg false discovery rate (FDR) correction. Pathways with an adjusted *p* value < 0.05 were considered statistically significant. Forest plots were generated to visualize the effect sizes and directions of associations across cancer types.

## 3 Results

### 3.1 Single‐Cell Transcriptome Atlas and Cell Typing in Polyp and Control Samples

**FIGURE 1:**
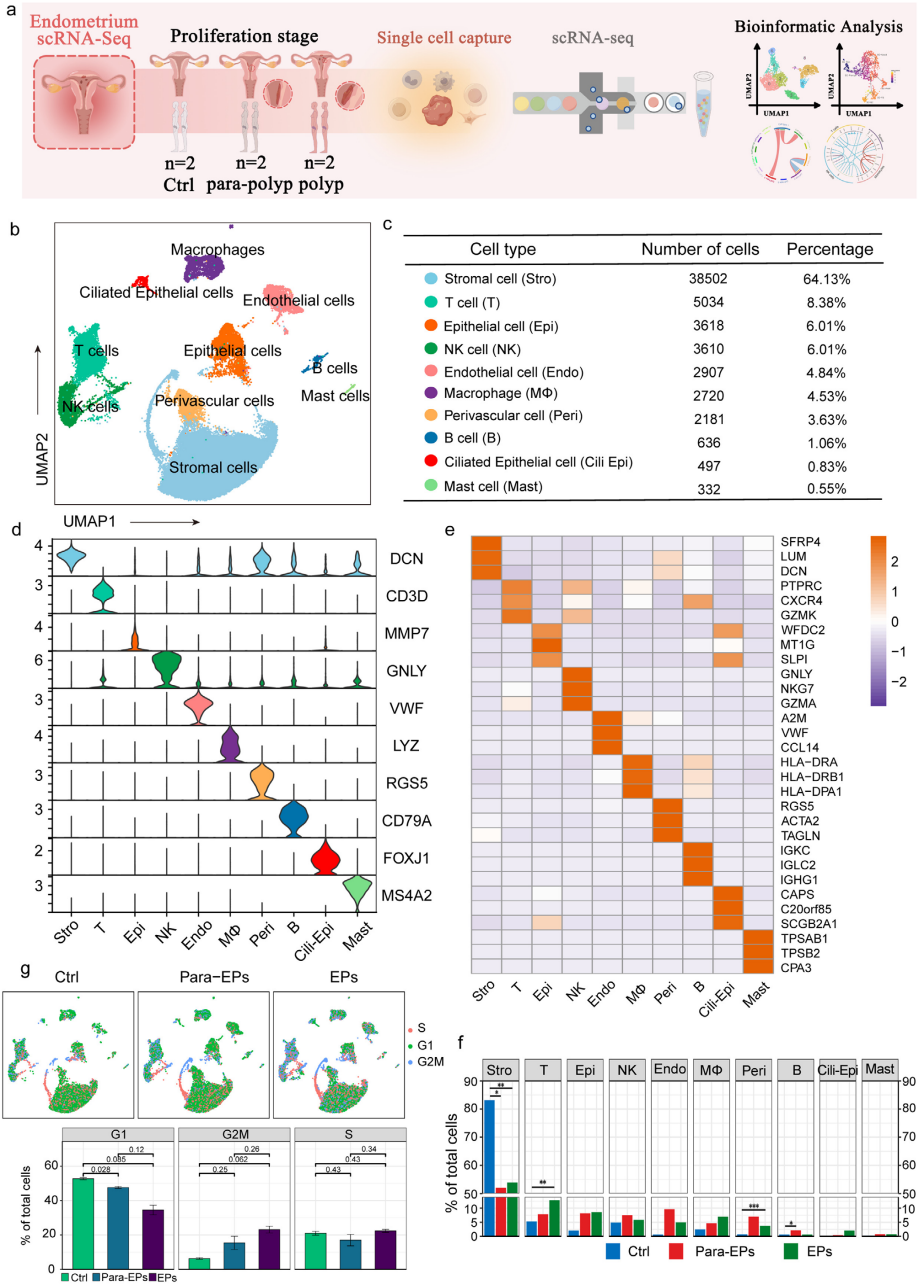
An overview of the single cell landscape of healthy controls and EPs patients. (a) A schematic outline illustrating the workflow for sample collection and integrated analysis, including the number of samples collected during the proliferative stage. (b) A Uniform Manifold Approximation and Projection (UMAP) plot displaying 10 distinct cell types identified in the scRNA‐seq data. Each point represents a single cell, color‐coded by cell type. (c) Chart showing the number and percentage of each cell type. (d) Violin plots displaying the expression levels of canonical markers for each cell type. (e) Heatmap showing the gene expression signatures of the top three marker genes for each cell type in the human endometrium. Column represents cell types; rows represent individual genes and shading indicates expression levels. (f) Bar plot showing the proportions of all cell types across the ctrl, para‐polyp, and polyp groups. (g) Cell cycle analysis of all cell types, displaying the proportion of cells in each phase across the control, para‐polyp, and polyp groups.

Endometrial tissues were collected from four patients with endometrial polyps and two healthy controls. The control samples were derived from our previously published dataset [21], which was generated using the same isolation and library preparation protocols to ensure technical consistency. Polyp tissues were categorized as para‐polyp or polyp based on sampling sites, with five samples collected during the proliferative phase of the menstrual cycle and one samples from secretory phase. Single‐cell RNA sequencing (scRNA‐seq) was then performed using 10× Genomics Chromium platform (Figure 1a). After quality control and cluster annotation, uniform manifold approximation and projection (UMAP) was applied to visualize clusters across 60 037 single cells. The results showed that control (CTRLl), para‐polyp, and polyp samples were all divided into 17 subpopulations (Figure S1a).

Based on previously reported canonical cell markers, 10 major cell types were identified (Figures S1b and 1b): epithelial cells (*EPCAM, CDH1*), ciliated epithelial cells (*FOXJ1, RFX2*), stromal cells (*DCN, LUM*), endothelial cells (*VWF, CDH5*), T cells (*CD3D, CD8A*), NK cells (*GNLY, GZMA*), mast cells (*MS4A2, CPA3*), B cells (*CD79A, IGKC*), macrophages (*CD14, CSF1R*), and perivascular cells (*ACTA2, RGS5*). The most abundant cell type was fibroblasts (64.13%), consistent with the typical cellular composition of mucosal tissues (Figure 1c). Finally, each identified cell type was characterized by unique marker genes (Figure 1d).

Next, we identified highly expressed genes in each cell type (Figure 1e) and performed gene ontology (GO) analysis, which further validated the accuracy of the cell type annotations (Figure S1c). The proportion of each cell type varied significantly across samples, indicating inter‐individual differences (Figure S1d). Notably, we observed distinct shifts in cell proportions across different cell types. Compared to normal endometrium, polyp and para‐polyp tissues exhibited a decrease in stromal cells and an increase in T cells and perivascular cells, suggesting alterations in immune status and enhanced vascularization in polyps (Figure 1f). Cell cycle analysis revealed that stromal cells, NK cells, and epithelial cells were primarily in the S and G2/M phases, while other cell types remained in the G1 phase, indicating increased cell proliferation in polyp tissues (Figure 1g). Collectively, our findings delineate the cellular composition of the endometrium and provide a comprehensive reference for further investigations into the pathophysiology of polyps.

### 3.2 Enhanced Estrogen Response, Epithelial Proliferation, and Inflammatory Signaling in Polyp Epithelium

**FIGURE 2:**
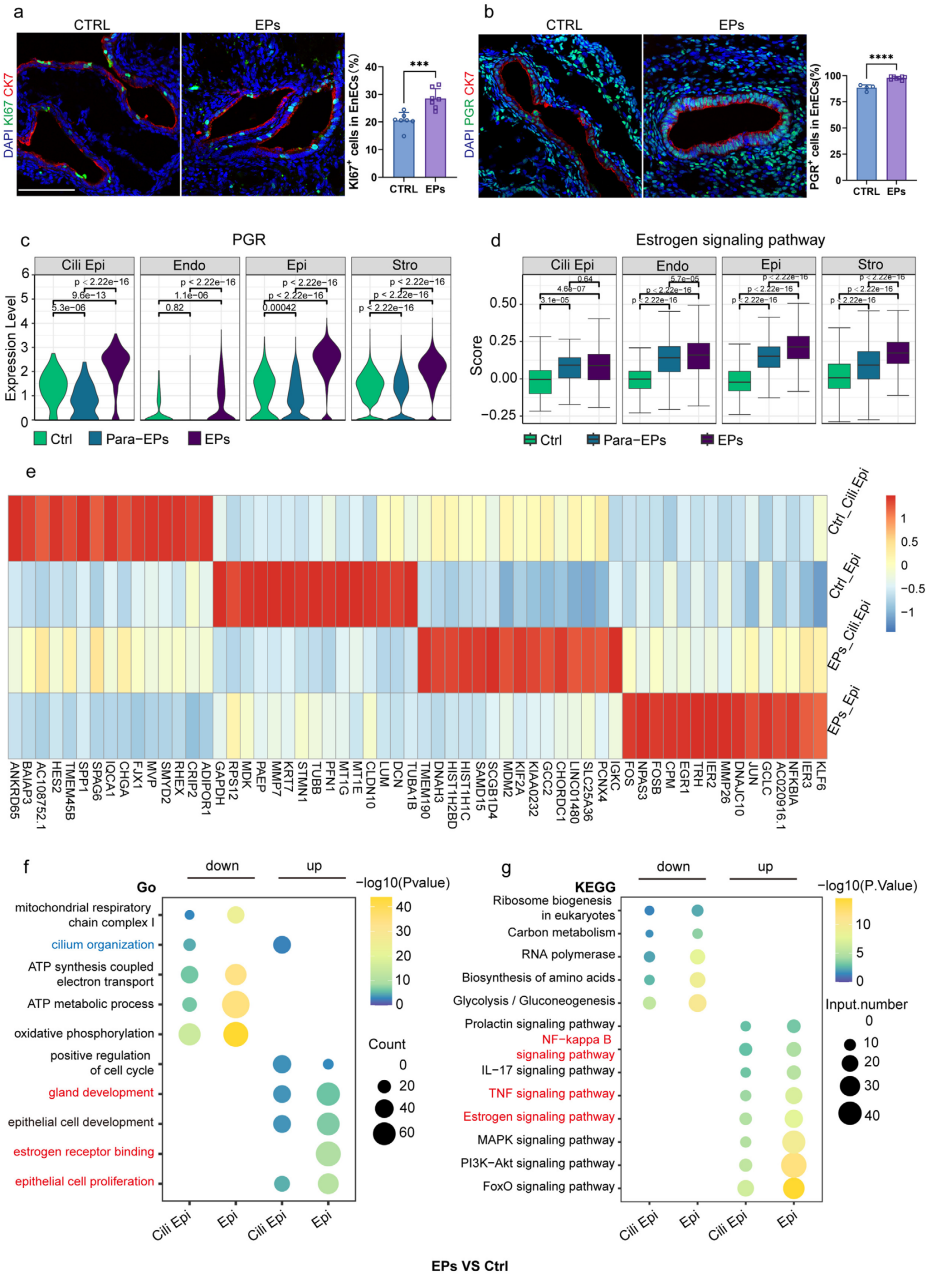
Pathological endometrial characteristics of EPs patients. (a, b) Representative immunofluorescent staining and quantification of KI67‐ and PGR‐positive epithelial cells in control endometrium (CTRL) and endometrial polyps (EPs). Data are presented as mean ± SEM. Statistical significance was determined by unpaired two‐tailed Student's *t*‐test. ***p < 0.001; ****p < 0.0001. Scale bar: 100 μm. (c) Violin plot showing PGR expression in ciliated, epithelial, endothelial and stromal cell type across the control, para‐polyp and polyp groups. (d) Violin plot showing enrichment scores for estrogen signaling pathways across different cell types in normal, para‐polyp, and polyp tissues. (e) Heatmap showing the expression of highly expressed genes in ciliated and non‐ciliated epithelial cell populations of EPs compared to normal endometrium. (f) Functional enrichment analysis of differentially expressed genes (DEGs) in ciliated epithelial (ciliated‐Epi) and epithelial (Epi) cells between the control (ctrl) and EPs groups. (g) KEGG enrichment analysis of upregulated and downregulated DEGs in ciliated‐Epi and Epi cells between the ctrl and EPs groups.

To determine whether an imbalance in the estrogen signaling pathway contributes to epithelial hyperplasia in endometrial polyps, we first analyzed the expression of PGR, a classical estrogen receptor (ER) target gene [28], across 10 endometrial cell types using UMAP (Figure S2a). Our analysis revealed that PGR was predominantly expressed in endometrial epithelial cells, stromal cells, and perivascular cells, with lower expression observed in immune cells (Figure S2b). Immunofluorescence and immunohistochemical analyzes of endometrial tissues revealed that epithelial cells in endometrial polyps exhibited a significantly higher proportion of PGR‐ and Ki67‐positive cells compared with normal endometrium, whereas epithelial ESR expression showed no significant difference between groups, indicating selective activation of progesterone‐associated proliferative programs rather than global steroid receptor upregulation. (Figures 2a,b and S2c–e).

To further investigate differences in PGR expression among non‐immune cell types, we analyzed ciliated epithelial cells, epithelial cells, stromal cells, and endothelial cells across the control, para‐polyp, and polyp groups. PGR expression was significantly higher in the polyp group compared with the control and para‐polyp groups (Figure 2c). Additionally, enrichment score analysis of estrogen‐related genes indicated an excessive estrogen response in structural cells within the polyp group (Figure 2d). Meanwhile, we observed significant enrichment of epithelial, ciliated epithelial, stromal, and endothelial cell clusters in the early estrogen response (Figure S3a). In addition, enrichment scores in the late estrogen‐responsive pathway showed enhanced epithelial, ciliated epithelial, stromal, and endothelial cell responses to estrogen (Figure S3b).

Next, we analyzed differential gene expression patterns in epithelial cell clusters to distinguish normal endometrium from both endometrial polyps and para‐polyps. The top 15 differentially expressed genes were visualized using a heatmap (Figures 2e and S3c). Functional enrichment and KEGG analysis of differentially expressed genes (DEGs) between normal and polyp endometrium revealed inhibition of oxidative phosphorylation, ATP synthesis/metabolic processes, and glycolysis/gluconeogenesis in epithelial cells, suggesting reduced cell proliferation (Figure 2f,g). In contrast, upregulated genes in polyp endometrium were enriched in pathways associated with “NF‐kappa B signaling”, “epithelial cell proliferation” and “estrogen signaling pathway,” indicating enhanced estrogen signaling and epithelial proliferation (Figure 2f,g). Similarly, compared with normal endometrium, para‐polyps exhibited significant enrichment in “epithelial cell proliferation,” “steroid hormone response,” and “NF‐kappa B signaling” (Figure S3d,e). Altogether, these findings suggest that epithelial cells in polyp endometrium exhibit reduced metabolic activity alongside increased proliferation and estrogen signaling activation compared with control samples.

### 3.3 T Cells With Low‐Cytotoxicity Signatures in Endometrial Polyp

**FIGURE 3:**
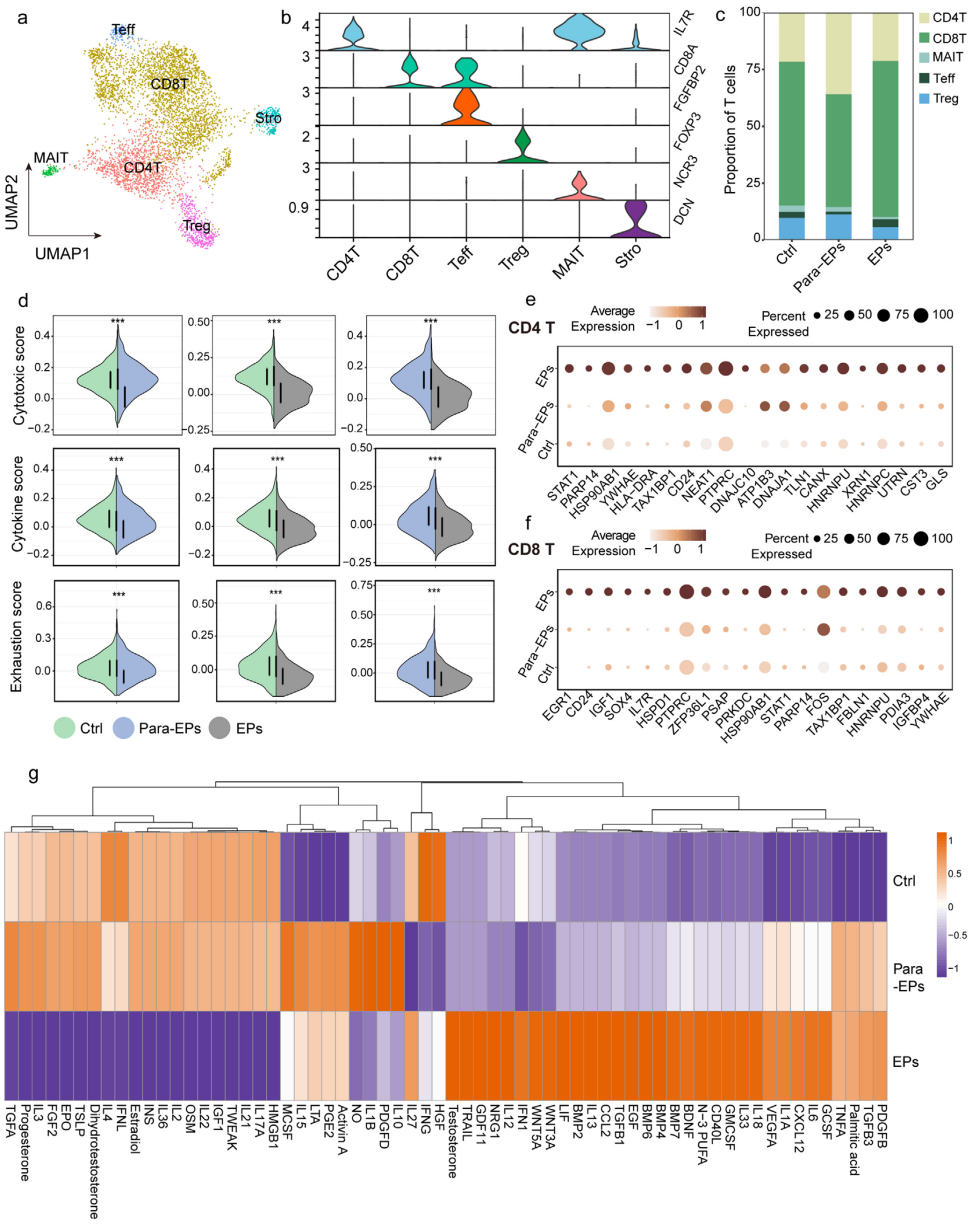
T cell subpopulations in endometrial polyp. (a) UMAP plot showing 5 T cells subpopulations identified, CD4T: CD4 T cell, Treg: Regulatory T cell, CD8T: CD8 T cell, MAIT: Mucosa‐associated invariant T, Teff: CD8 effector T cell. (b) Canonical gene markers for each T cells subpopulation. (c) overall proportions of T cells subpopulations (color legend) according to Ctrl, para‐EPs, EPs. (d) Violin plot showing cytotoxic, cytokine, exhaustion scores of T cells in Ctrl and endometrial polyp subtypes (para‐EPs, EPs). (e, f) Dot plot showing DEG markers of T cell subpopulations (CD4T and CD8T) according to ctrl, para‐EPs, EPs tissues. (g) Heatmap showing DEG cytokine and growth factors according to ctrl, para‐EPs, EPs tissues.

Recent studies have shown that chronic endometritis increases the risk of polyp formation [29]. To investigate the relationship between inflammation and polyps, we analyzed the immune status of T cells in polyp tissues. We identified 5 T‐cell subtypes: CD4, CD8, Treg, MAIT, and Teff (Figure 3a,b). A group of stromal cells was mistakenly identified as T cells and was excluded from subsequent analyzes (Figures 3c and S4a–d). The proportions of these 5 T cell subtypes did not differ significantly among the control, para‐polyp, and polyp groups (Figure 3c). Therefore, we further analyzed their functional status.

The cytotoxicity, cytokine, and exhaustion scores of T cells were lower in the para‐polyp and polyp groups than in the control group (Figure 3d). Notably, STAT1, an inflammatory transcription factor [30], was highly expressed in both CD4 and CD8 T cells in the polyp group (Figure 3e,f). To better characterize the inflammatory environment within polyps, we used a heatmap to visualize differentially expressed genes, including growth factors and cytokines. Polyps exhibited high expression of inflammatory factors, including IL‐12, IL‐18, IL‐1A, IL‐6, and GM‐CSF, whereas the para‐polyp and control groups predominantly expressed IL‐15, IL‐1β, M‐CSF, and IL‐22 (Figure 3g). These findings suggest that T cell immune transformation may contribute to polyp formation.

### 3.4 Genomic Alterations and Transcriptional Regulation in Polyp Epithelial Subpopulations

**FIGURE 4:**
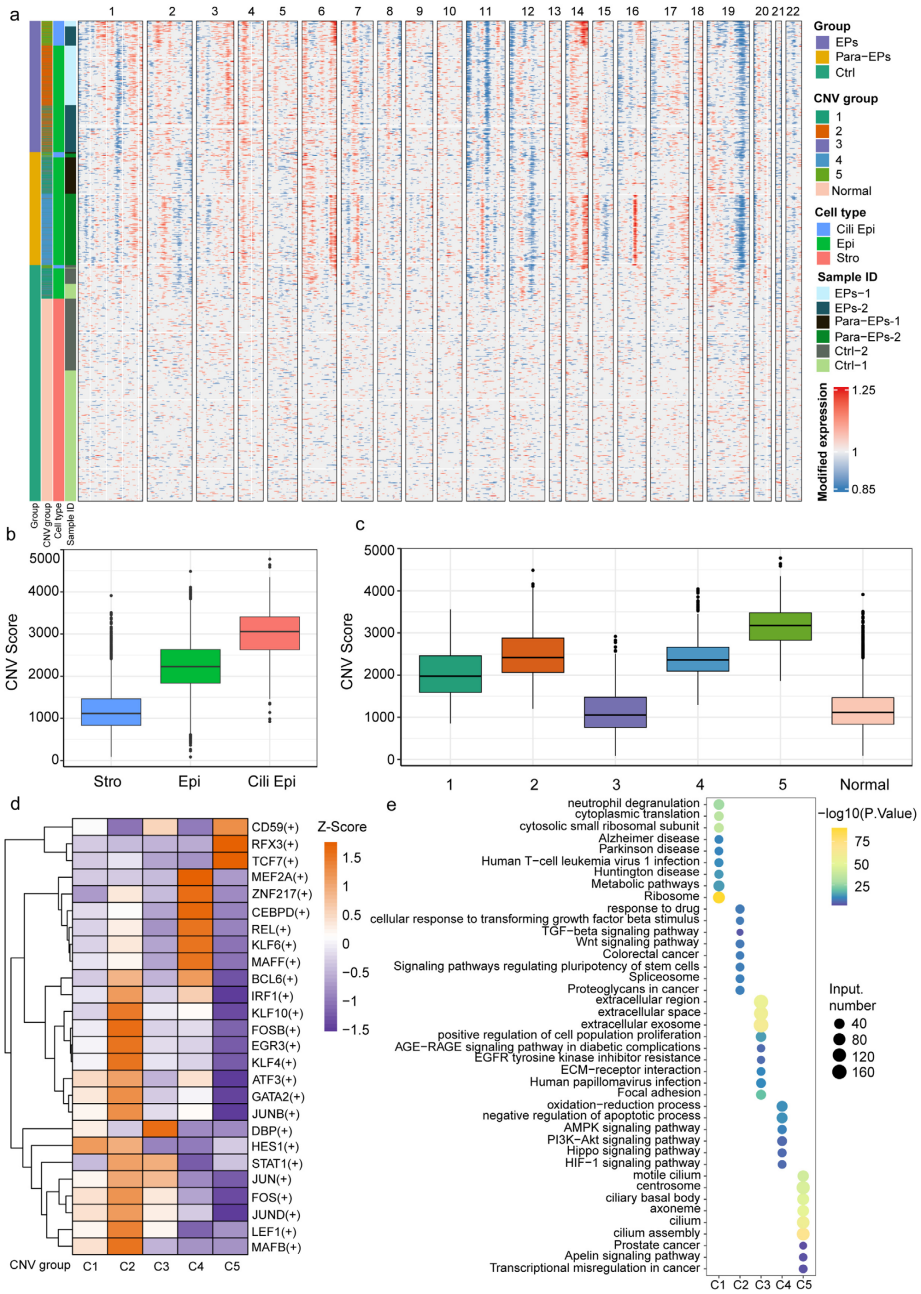
Molecular differences among epithelial cell subclusters. (a) Inferred large‐scale CNA levels in each epithelial cell cluster across 22 chromosomes; red indicates a high CNV level, and blue indicates a low CNV level. (b) Copy number variation score across different endometrial cell types. (c) Comparisons of overall CNA levels among different epithelial subclusters. (d) Heatmap shows the relative activity scores of biological processes in each epithelial subcluster. (e) Heatmap shows the relative expression of differential transcription factors in each epithelial cell subcluster.

Although most endometrial polyps are benign, we aimed to investigate the underlying causes of early epithelial hyperplasia by analyzing copy number variations (CNVs) in epithelial cells based on their gene expression profiles. Since CNVs are relatively low in normal endometrial stromal cells, we used them as a baseline for comparison among the control, EP, and para‐EP groups (Figure 4a,b). By examining large‐scale chromosomal CNVs inferred from transcriptomic data, we analyzed single‐cell CNVs in epithelial cells from these groups. Our findings revealed frequent CNVs on chromosomes 3, 4, 14, and 16, suggesting that these chromosomal alterations may represent typical CNV subclones contributing to polyp formation (Figure 4a).

The epithelial cells were further divided into five clusters with distinct CNV levels. Clusters 2 and 5 exhibited high CNV levels, clusters 1 and 4 showed medium CNV levels, and cluster 3 displayed low CNV levels (Figure 4c). Functional enrichment analysis revealed that genes highly expressed in subcluster 5 were associated with cilium assembly, the centrosome, and the ciliary basal body. Clusters 1 and 2 were enriched in ribosome components, metabolic pathways, and cytoplasmic translation, whereas cluster 3 was related to extracellular regions, extracellular spaces, and extracellular exosomes. Cluster 4 was associated with HIF, PI3K‐Akt, and AMPK signaling pathways (Figure 4d).

To further investigate transcriptional regulation in proliferative epithelial subclusters of endometrial polyps, we applied the single‐cell regulatory network inference and clustering (SCENIC) method. Our analysis identified multiple transcription factors with key regulatory roles, including HES1 in subcluster C1; DBP in subcluster C3; ZNF217, CEBPD, and KLF6 in subcluster C4; TCF7, CD59, and RFX3 in subcluster C5; and IRF1, KLF10, FOS, JUN, JUND, LEF1, EGR3, STAT1 in subcluster 2 (Figure 4e). In summary, our findings highlight the genomic and transcriptomic heterogeneity among epithelial subpopulations.

### 3.5 WNT Signaling Interactions Between Stromal and Epithelial Cells Are Altered in Endometrial Polyps

Endometrial polyps are often accompanied by glandular hyperplasia, characterized by dysregulated epithelial proliferation. As Wnt signaling is known to participate in estrogen‐driven epithelial proliferation [31], we further explored whether altered Wnt‐mediated communication between epithelial and stromal cells may contribute to the pathogenesis of polyps.

To characterize stromal cell heterogeneity, we performed UMAP‐based dimensionality reduction followed by unsupervised clustering, identifying eight stromal subpopulations (S0–S7) and highlighting marked cellular heterogeneity (Figure S5a–c). Next, we mapped our epithelial cells to the epithelial cell types defined in the Human Endometrial Cell Atlas (HECA) [32], identifying three major subtypes: SOX9^+^, ciliated and glandular epithelial cells (Figure S5d). Notably, the glandular subtype was detected in only one sample from the para‐EPs group, which may be attributed to the sample being in the secretory phase (Figure S5e).

**FIGURE 5:**
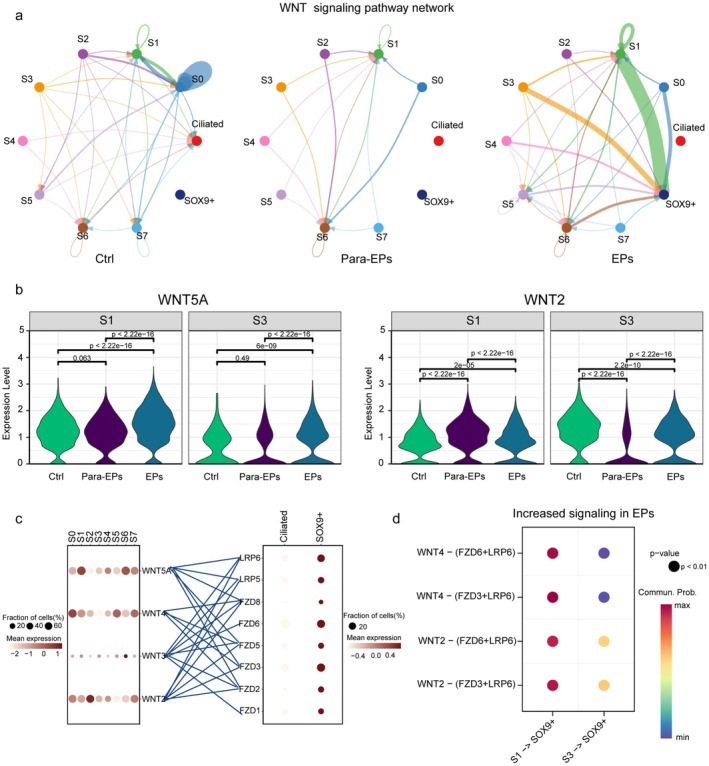
Enhanced WNT signaling interactions between epithelial and stromal cells in EPs. (a) Inferred cell–cell communication networks via the WNT signaling pathway among epithelial and stromal subpopulations in control (Ctrl), para‐EPs, and EPs groups. The strength and direction of intercellular signaling are visualized with colored and weighted edges. (b) Violin plots showing expression levels of WNT5A and WNT2 in stromal clusters S1 and S3 across Ctrl, para‐EPs, and EPs samples. Statistical significance between different groups was evaluated with the two‐sided Wilcoxon rank sum test. (c) Expression profiles of WNT signaling components (ligands, receptors, and co‐receptors) across epithelial and stromal clusters. (d) Bubble plot showing predicted WNT ligand‐receptor interactions that are significantly upregulated in the EPs group, from S1 and S3 stromal subtypes to SOX9^+^epithelial cells. Bubble size indicates statistical significance (*p* < 0.01), and color represents the communication probability.

To ensure consistency in downstream analyzes, Wnt signaling interactions were assessed only between the two epithelial subtypes (SOX9^+^ and ciliated) and the eight stromal subpopulations (S0–S7). To comprehensively investigate the incoming and outgoing signaling between stromal and epithelial subpopulations in the endometrium, we employed CellChat to analyze intercellular communication patterns. Cell types with similar signaling enrichment profiles were clustered on the left, while the corresponding signaling molecules enriched in each pattern were displayed on the right (Figure S6a). WNT signaling emerged as a central component in stromal cell communication networks across both incoming and outgoing directions (Figure S6a). Cell–cell communication analysis revealed that Wnt signaling was predominantly active within the S0 stromal subset in the control group. In contrast, stromal‐epithelial Wnt signaling was lost in the para‐EPs group. In the EPs group, aberrantly elevated Wnt signaling was observed between the stromal subsets (S1, S3) and SOX9^+^ epithelial cells, a population known to expand during the proliferative phase (Figure 5a). These results suggest that the abnormal proliferation of glandular epithelial cells in endometrial polyps may be driven by enhanced Wnt‐mediated stromal‐epithelial interactions. Notably, WNT2 and WNT5A expression in the S1 and S3 stromal subsets was elevated in the EPs group compared to both the Ctrl and para‐EPs groups (Figure 5b).

WNT signaling analysis revealed signals from fibroblast clusters S0‐S7 to ciliated epithelial cells (Figure 5a). This directionality was supported by the expression patterns of WNT ligands in stromal clusters (S0‐S7) and their receptors (FZD3, FZD6, LRP) in epithelial clusters (Figures 5c and S5f). Notably, WNT2, WNT4, and WNT5A were prominently expressed in stromal subsets, while FZD3 and FZD6 were the main receptors in epithelial cells (Figure 5c). CellChat analysis of differential ligand‐receptor interactions revealed consistent upregulation of WNT2 and WNT4 and their corresponding receptors (FZD3, FZD6, LRP6) in the EPs group (Figure 5d). Additionally, we presented the top ten differentially expressed genes in five immune cell types—T cells, B cells, NK cells, macrophages, and mast cells—in both the Para‐EPs vs. Ctrl and EPs vs. Ctrl comparisons (Figure S6b,c). Together, our scRNA‐seq data highlight a key role for specific fibroblast subpopulations in the endometrium and polyps, suggesting that stromal‐epithelial WNT signaling may contribute to epithelial hyperplasia and the pathogenesis of endometrial polyps.

### 3.6 Altered Stromal‐Centric Intercellular Signaling Networks in Endometrial Polyps Revealed by CellChat Analysis

**FIGURE 6:**
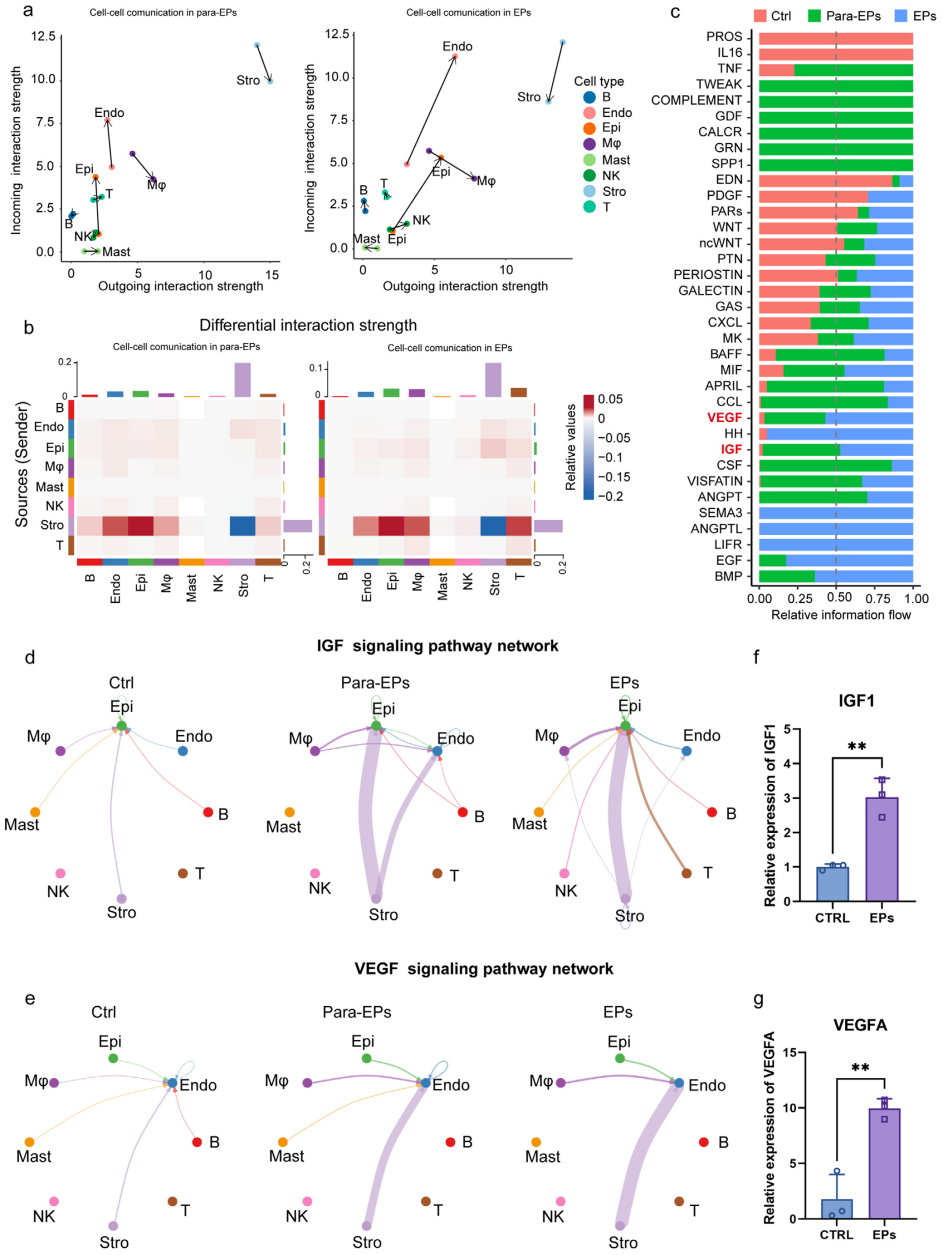
Cell‐Chat analysis reveals altered intercellular communication patterns in para‐EPs and EPs. (a) Arrow plots illustrating changes in outgoing and incoming interaction strengths between para‐EPs (arrow tip) and control (arrow base) (left), and between EPs (arrow tip) and control (arrow base) (right) across major cell types. (b) Heatmaps showing differential interaction strengths between control and para‐EPs (left), and between control and EPs (right). Red and blue indicate increased and decreased signaling interactions, respectively. (c) Bar plot showing the relative information flow of major signaling pathways in ctrl, para‐EPs, and EPs groups. Communication strength was calculated by aggregating all predicted communication probabilities within each pathway. (d, e) Circle plots depicting intercellular signaling interactions involved in the IGF pathway (d) and VEGF pathway (e) across control, para‐EPs, and EPs groups. Nodes represent distinct cell types; edge thickness indicates interaction strength. (f‐g) Quantitative RT‐PCR analysis of IGF1 (f) and VEGFA (g) transcript level in control (CTRL; circles) and endometrial polyp (EPs; squares) groups. Data are shown as mean ± SEM. Statistical significance was assessed by an unpaired two‐tailed Student's *t*‐test, with *p* < 0.05 considered statistically significant.

Tissue development and the progression of complex diseases depend on intricate networks of intercellular communication. To explore these dynamics in the context of endometrial polyps, we applied CellChat analysis. We first visualized the incoming and outgoing signaling patterns of all cell types across the Ctrl, para‐EPs and EPs groups (Figure S7a,b). The direction and length of each arrow represent the dominant signaling direction and relative interaction strength associated with polyp‐specific alterations (Figure 6a). Incoming signals to stromal cells showed a pronounced decrease in both the para‐EPs and EPs groups, while their outgoing signals exhibited a slight reduction specifically in the EPs group. In contrast, outgoing signals from epithelial cells, endothelial cells, and macrophages were significantly elevated in the EPs group. (Figure 6a). We next compared intercellular interaction strengths across all cell types in the para‐EPs vs. Ctrl and EPs vs. Ctrl groups (Figure 6b). In both cases, stromal‐stromal interactions were reduced, while interactions from stromal cells (as senders) to endothelial cells, epithelial cells, macrophages, and T cells (as receivers) were notably increased (Figure 6b).

To further dissect the molecular basis of these changes, we examined the specific signaling pathways underlying the altered cellular interactions across the Ctrl, para‐EPs, and EPs groups. In the EPs group, the most significantly upregulated pathways included SEMA3, ANGPTL, and LIFR; these pathways were almost completely inactive in the control group but became highly active in the EPs group, whereas the EDN and PARs pathways showed the most pronounced reductions in signaling strength (Figure 6c). The communication strength of the VEGF and IGF pathways was significantly elevated in the EPs group, and was particularly notable (Figure 6c). Both IGF and VEGF signals primarily originated from stromal cells. IGF receptors were mainly expressed by epithelial cells, whereas VEGF receptors were predominantly expressed on endothelial cells (Figure 6d,e). Quantitative PCR analysis confirmed that both IGF1 and VEGFA expression levels were significantly increased in endometrial polyps compared with normal endometrium, supporting enhanced stromal‐derived IGF and VEGF signaling in EPs (Figure 6f,g).

In summary, the intercellular interactions within the endometrium exhibited alterations consistent with the transcriptomic findings. These changes may indicate a shift in stromal cell function from providing support to promoting polyp‐associated signaling, possibly driven by overactive interactions among stromal and epithelial cells, leading to excessive proliferation. These findings highlight the central role of stromal cells in coordinating endometrial signaling and suggest that disrupted stromal communication may contribute to EPs development.

### 3.7 Validation of Enhanced Epithelial‐Stromal WNT Signaling in Spatial Transcriptomics and Single‐Cell Datasets

**FIGURE 7:**
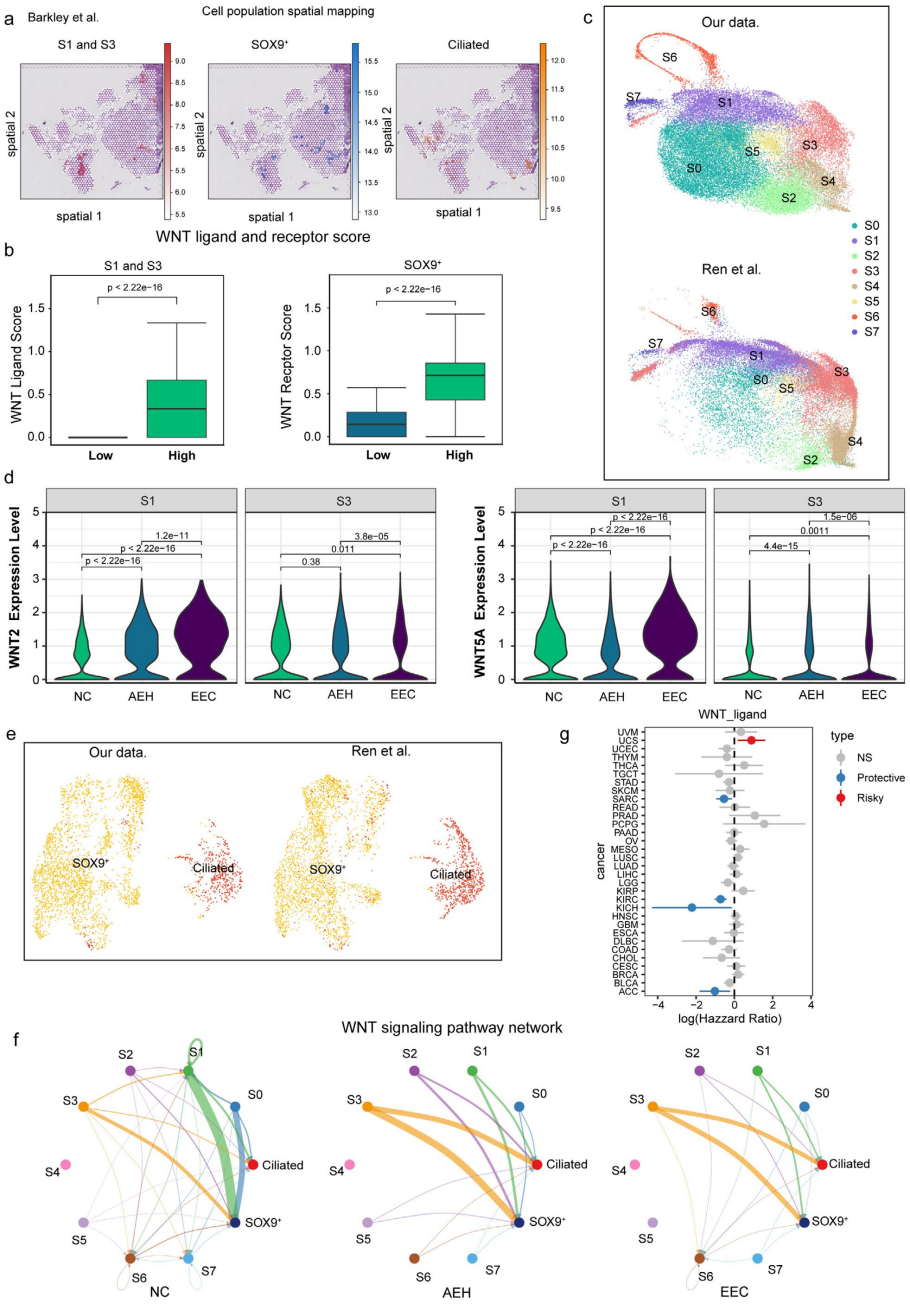
Cell type mapping in endometrial carcinoma tissue slice and cell–cell communication validation in single‐cell data. (a) Spatial mapping of stromal subclusters (S1 and S3), SOX9^+^epithelial cells, and ciliated epithelial cells in the spatial transcriptomic dataset from Barkley et al. (b) Comparison of WNT ligand and receptor expression scores between regions with high and low enrichment of S1 + S3 stromal or SOX9^+^ epithelial subclusters. (c) Integration of stromal subclusters from our dataset and Ren et al. showing consistent subcluster distribution. (d) Violin plots showing expression of WNT2 and WNT5A across normal endometrium (NC), atypical endometrial hyperplasia (AEH), and endometrioid endometrial carcinoma (EEC) within stromal subclusters S1 and S3. (e) Mapping of epithelial subclusters (SOX9^+^ and ciliated) from both datasets demonstrating similar spatial distribution patterns. (f) WNT signaling interaction networks between epithelial and stromal subclusters across three groups. (g) Forest plot depicting log‐transformed hazard ratios (log HR) of WNT ligand expression for overall survival across TCGA cancer types. Each dot represents the log HR for an individual cancer type, with horizontal lines denoting 95% confidence intervals. Dots are colored based on prognostic significance: Gray (not significant, NS), blue (protective, HR < 1, *p* < 0.05), and red (risky, HR > 1, *p* < 0.05).

Building on our single‐cell findings that enhanced WNT signaling between epithelial and stromal cells may contribute to polyp development, we further validated this interaction in Barkley et al.’ endometrial cancer spatial transcriptomic data [33]. Stromal subclusters S1 and S3 were spatially co‐localized with epithelial subclusters SOX9^+^ and ciliated cells (Figure 7a). Moreover, regions with high S1 + S3 and SOX9^+^ enrichment exhibited higher expression scores of WNT ligands and receptors, respectively, than regions with low subcluster localization (Figure 7b).

To further validate epithelial‐stromal WNT signaling, we examined the single‐cell dataset of endometrial cancer from Ren et al. [34]. We first defined 10 major cell types‐epithelial, ciliated epithelial, stromal, endothelial, T cells, B cells, NK cells, macrophages, mast cells, and pericytes‐and showed their proportions among normal endometrium (NC), atypical endometrial hyperplasia (AEH), and endometrioid endometrial carcinoma (EEC) (Figure S8a,b). Stromal cells from this dataset were then mapped onto our own dataset (Figure 7c). During malignant progression, stromal expression of WNT2, WNT4, and WNT5A gradually decreased (Figure S8c). However, consistent with our findings in endometrial polyps, WNT2 and WNT5A expression remained elevated in stromal subclusters S1 and S3 (Figure 7d).

Likewise, mapping epithelial cells from the Ren et al. dataset to ours (Figure 7e) revealed enhanced WNT signaling interactions between stromal subcluster S3 and the SOX9^+^ epithelial subcluster (Figure 7f). To evaluate the potential prognostic relevance of dysregulated WNT signaling, we further examined the association between WNT ligand expression and patient survival using publicly available cancer datasets. Pan‐cancer survival analysis revealed that increased expression of WNT ligands was significantly associated with poorer overall survival in uterine carcinosarcoma, suggesting that WNT signaling plays a context‐dependent yet clinically relevant role in uterine malignancies (Figure 7g). Together, these results suggest that enhanced WNT signaling between stromal and epithelial cells plays a critical role in the initiation and progression of endometrial polyps.

### 3.8 Transcriptomic Alterations in Epithelial Cells Also Contribute to Endometrial Polyp Development

**FIGURE 8:**
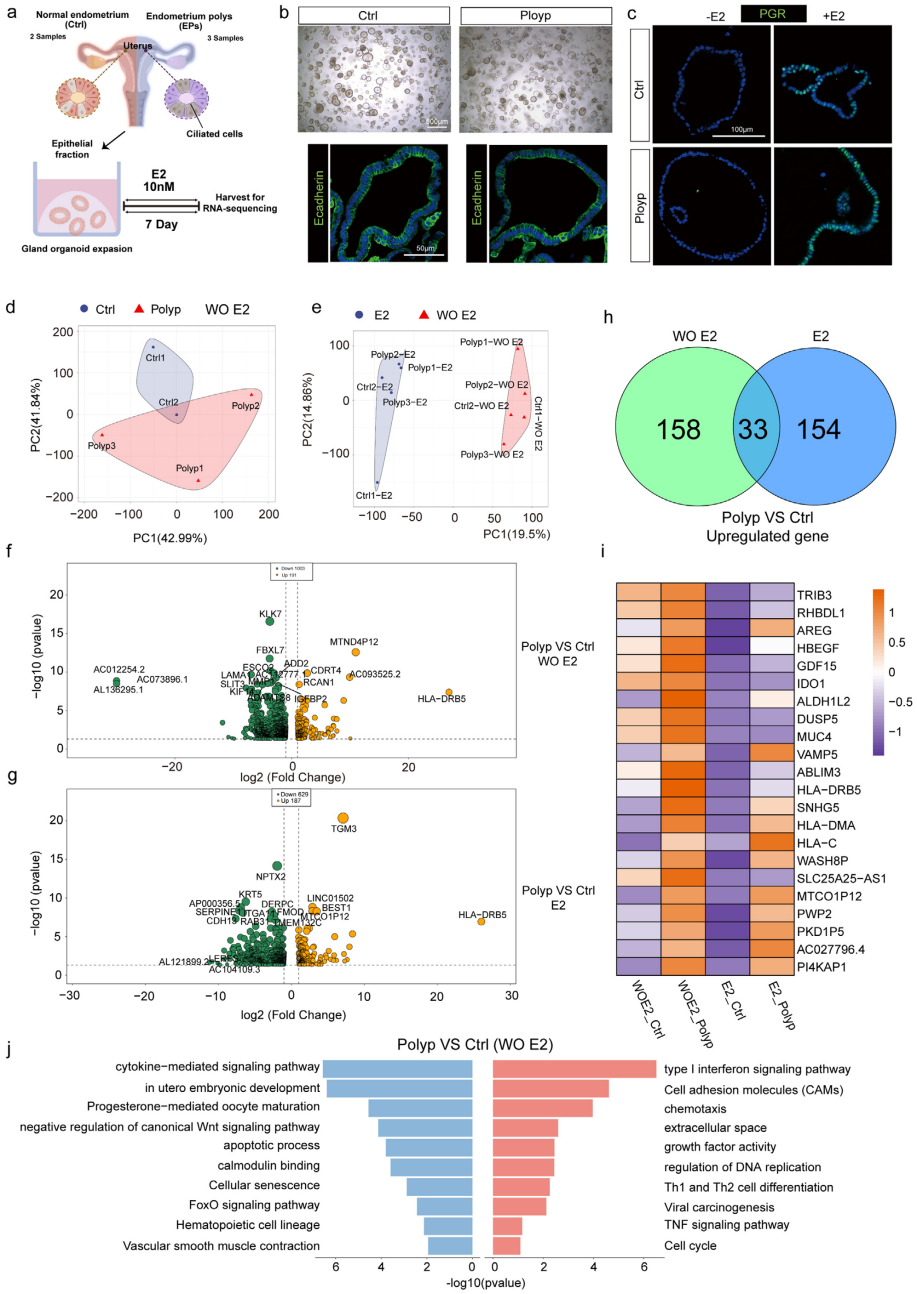
Transcriptomic alterations in epithelial cells. (a) Schematic overview of the experimental design. Endometrial tissues from healthy controls (Ctrl) and polyp patients (Polyp) were collected, epithelial fractions were isolated to establish glandular organoids, and organoids were treated with estradiol (E2, 10 nM) for 7 days to mimic proliferative‐phase conditions before bulk RNA sequencing. (b) Representative bright‐field and immunofluorescence images of glandular organoids derived from Ctrl and Polyp endometrial tissues, showing comparable morphology and expression of epithelial marker EPCAM (green). Scale bars, 500 μm (upper), 50 μm (bottom). (c) Immunofluorescence staining showing the expression of progesterone receptor (PGR, green) in control and polyp organoids under untreated (−E2) and E2‐treated (+E2) conditions, indicating hormone responsiveness. Nuclei were counterstained with DAPI (blue). Scale bars, 100 μm. (d, e) Principal component analysis (PCA) for bulk‐RNA sequencing data of tissue samples from polyp and control groups under E2 treatment or untreated conditions. (f, g) Volcano plots showing genes with log2(FC) on the horizontal axis and −log10(*p*‐value) on the vertical axis. FC, fold change of polyps relative to controls. (h) Venn diagram showing the overlap of upregulated genes in polyps relative to controls under both conditions. (i) Heatmap displaying the top 22 genes upregulated in polyps under both E2‐treated and untreated conditions relative to controls. (j) KEGG and GO enrichment analyzes of differentially expressed genes, with blue representing down‐regulation and red representing up‐regulation.

Although aberrant WNT interactions between stromal and epithelial cells may contribute to the development of endometrial polyps, transcriptional alterations within epithelial cells themselves may also play a pivotal role. To further investigate epithelial cell changes during the proliferative phase, we collected endometrial tissues from patients with polyps (Polyp) and healthy controls (Ctrl), isolated glandular epithelial cells, and cultured them as organoids in vitro as previously described [26]. Organoids were treated with estradiol (E2) for 7 days to mimic proliferative‐phase conditions before bulk RNA sequencing (Figure 8a).

Organoids derived from both control and polyp endometrial tissues exhibited typical glandular morphology under bright‐field microscopy, forming spherical and cystic structures with a single epithelial layer (Figure 8b). Immunofluorescence staining confirmed the epithelial identity of these organoids, as indicated by strong EPCAM expression along the apical surface. Furthermore, the expression of progesterone receptor (PGR) was markedly induced upon E2 stimulation in both control and polyp organoids, indicating that the established organoid model retained hormone responsiveness and faithfully recapitulated proliferative‐phase characteristics (Figure 8c).

Principal component analysis (PCA) revealed clear segregation between normal and polyp‐derived glandular organoids (Figures 8d and S9a,b). Similarly, E2‐treated and untreated groups clustered separately (Figure 8e), indicating distinct transcriptomic states in polyp tissues compared with controls, regardless of estrogen treatment. Volcano plots showed that, under non‐E2 conditions, 191 genes were significantly upregulated and 1003 genes were downregulated in polyps compared with controls (Figure 8f). Under E2 treatment, 187 genes were significantly upregulated and 629 genes were downregulated (Figure 8g).

We next identified the intersection of upregulated genes between E2‐treated and untreated polyps compared with controls (Figure 8h), yielding 33 common upregulated genes, with the top 22 displayed in the heatmap (Figure 8i). Similarly, 255 genes were commonly downregulated, and the top 22 were visualized in a heatmap (Figure S9c,d). Notably, KEGG and GO enrichment analyzes of upregulated genes—both with and without E2 treatment—showed significant enrichment in cell adhesion molecules (CAMs), growth factor activity, Th1 and Th2 cell differentiation, inflammatory response, and NF‐κB signaling pathways. In contrast, genes downregulated under both conditions were enriched in the negative regulation of the canonical WNT signaling pathway, while enrichment in the apoptosis process was observed only in the absence of E2 stimulation (Figures 8j and S9e).

Collectively, these findings suggest that endometrial polyp formation is driven not only by disrupted stromal‐epithelial interactions but also by intrinsic transcriptional reprogramming of epithelial cells, which may further promote lesion development.

## 4 Discussion

In this study, we present a comprehensive single‐cell transcriptomic analysis of EPs, revealing significant cellular, molecular, and intercellular communication alterations that may underlie polyp formation and progression. By profiling over 60 000 single cells from control, para‐EPs, and EPs tissues, we uncovered profound changes in cellular composition, estrogen signaling, immune cell status, and stromal‐epithelial interactions that collectively illuminate the pathophysiology of EPs.

Our findings demonstrate a marked increase in epithelial proliferation and estrogen responsiveness in endometrial polyps, supporting the notion that an aberrant estrogen microenvironment promotes epithelial overgrowth. Specifically, we observed elevated expression of PGR and enrichment of estrogen‐responsive gene sets in epithelial, stromal, and perivascular cells in polyp tissues. In parallel, functional analysis of differentially expressed genes revealed activation of the NF‐κB pathway in epithelial cells, suggesting a pro‐inflammatory component to this hyperproliferative state. Furthermore, immune profiling revealed that T cells in polyps exhibited reduced cytotoxic and cytokine activity, yet expressed higher levels of STAT1 and pro‐inflammatory cytokines such as IL‐6, IL‐1A, and GM‐CSF. These features point to a chronic, low‐grade inflammatory microenvironment that may simultaneously drive epithelial proliferation and impair immune surveillance. Such immunosuppressive conditions may underlie the occasional malignant transformation observed in EPs, particularly among postmenopausal women.

The WNT signaling pathway has been extensively studied in the context of adenomyosis [35], endometrial regeneration [36], and endometrial cancer [37]. It is well established that during the proliferative phase of the menstrual cycle, rising estrogen levels activate WNT signaling, thereby promoting the growth and thickening of the endometrium [38]. Most studies have focused on epithelial cell‐intrinsic WNT activity and its influence on epithelial behavior [39], while little is known about whether niche (stromal) cells also exhibit active WNT signaling and how this may affect stem or progenitor cell function. Moreover, the cell type‐specific roles of WNT signaling in the context of EPs remain poorly defined. In this study, we identified an enhanced WNT signaling network between stromal subpopulation S1 and a SOX9^+^ epithelial subset, the latter representing the major proliferative epithelial compartment in the endometrium [40]. In particular, WNT2 and WNT5A ligands were markedly upregulated in stromal subsets (S1 and S3), while their receptors (FZD3, FZD6, and LRP6) were enriched in SOX9^+^ epithelial cells. Moreover, the aberrant interaction between SOX9^+^ epithelial cells and S3 stromal subsets was further confirmed in endometrial carcinoma spatial transcriptomic data, based on their spatial colocalization and enhanced WNT signaling communication.

These findings suggest a reorganization of WNT signaling between epithelial and stromal compartments within EP tissues, potentially driving epithelial hyperplasia and polyp formation. Similarly, stromal secretion of IGF and VEGF likely supports epithelial growth and angiogenesis, respectively, further reinforcing the central role of stromal cells as key regulators of polyp development.

Currently, there are limited studies on the pathogenesis of EPs. One study highlights mast cell‐driven inflammation and WT1 dysregulation as key factors contributing to EPs development [22]. Another study systematically analyzed the mutational landscape of tumor‐related genes in EPs and concluded that these lesions are non‐clonal. However, it proposed that the progression of EPs may result from the age‐related accumulation of oncogenic mutations within polyps, allowing them to evade normal menstrual shedding [14]. In our study, we inferred CNVs in epithelial cells. The presence of CNV‐high epithelial subsets, particularly those enriched in cilium assembly and estrogen signaling pathways, suggests that early genomic instability may drive clonal expansion and hyperplasia in a subset of EPs. SCENIC analysis revealed subtype‐specific transcriptional regulators, including HES1, ZNF217, STAT1, and TCF7, that may orchestrate epithelial reprogramming and proliferation. TCF7 and HES1 were previously reported to regulate cell proliferation [41, 42]. Further studies on the mechanisms of EP formation may offer important clues for advancing endometrial regeneration strategies.

Furthermore, in our study, we established endometrial glandular organoids derived from normal and polyp tissues to simulate the proliferative phase in vitro by E2 treatment, revealing that intrinsic transcriptional alterations in epithelial cells also contribute to polyp formation. In the future, leveraging our previously developed endometrial assembloid model containing luminal epithelium [43], we plan to further investigate epithelial‐stromal interactions involved in polyp development and explore the molecular dynamics of polyps across the menstrual cycle, thereby providing a more comprehensive understanding of the pathogenesis of endometrial polyps.

Collectively, our findings reveal that endometrial polyps are not simply localized overgrowths but represent a complex ecosystem of transcriptionally reprogrammed epithelial and stromal cells, embedded in a dysregulated immune and hormonal environment. Our data suggest that stromal cells act as a central hub integrating estrogenic, inflammatory, and paracrine signals that fuel epithelial proliferation and structural remodeling. Targeting the stromal‐epithelial axis, or restoring immune and hormonal balance, may represent promising therapeutic strategies for the prevention or treatment of recurrent EPs.

Future studies integrating spatial transcriptomics and epigenetic profiling may further elucidate the microenvironmental context and origin of key stromal subtypes involved in polyp formation. Moreover, longitudinal profiling of polyp evolution could shed light on the transition from benign hyperplasia to potential malignant transformation. We also acknowledge that the relatively small number of patient samples in this study may limit the generalizability of our findings and may not fully capture the heterogeneity of endometrial polyps across different populations. Future studies with larger and more clinically diverse cohorts will be important to validate and extend our observations. Together, our work establishes a valuable single‐cell atlas of EPs and provides mechanistic insights into its pathogenesis.

## Author Contributions

Conceived and designed experiments: E. Dong and Tianqing Li. Performed the experiments: E. Dong, Zhengli Zhou, Bo Zhang, Xiaozhuo Li, Huimei Zhang, and Ting Liu. Collected clinical patients' samples: E. Dong, Zhengli Zhou, Xiaomei Wu, Tao Yu, and Xiaodie Wang. Analyzed the bioinformatic data: E. Dong, Tingwei Chen, Naixue Yang, and Yu Yin. Wrote the manuscript: E. Dong. Revised the article: Tianqing Li and E. Dong. All authors read and approved the final version of the manuscript.

## Funding

This work was supported by the National Natural Science Foundation of China (Grants 32560179, 32130034, and 82192874), the National Key Research and Development Program of China (Grant 2022YFA1103100), Central Government Guidance Fund for Local Science and Technology Development (Grant 202407AB110013), Yunnan Fundamental Research Projects (Grants 202201AU070232, 202501AT070168, and 202401CF070089) and the Yunnan Revitalization Talent Support Program.

## Ethics Statement

This study was approved by the Ethics Committee of First People's Hospital of Yunnan Province, affiliated with Kunming University of Science and Technology (ethics number: KHLL2021‐KY049) and has been performed in accordance with the principles of the Declaration of Helsinki.

## Consent

Written informed consent was obtained from all patients in this study. All authors have given their consent to publish.

## Conflicts of Interest

The authors declare no conflicts of interest.

## Supporting information


**Data S1:** fsb271645‐sup‐0001‐Supinfo.docx.

## Data Availability

The raw sequence data reported in this paper have been deposited in the Genome Sequence Archive (Genomics, Proteomics & Bioinformatics 2025) in the National Genomics Data Center (Nucleic Acids Res 2025), China National Center for Bioinformation/Beijing Institute of Genomics, Chinese Academy of Sciences (GSA‐Human: HRA013826) that are publicly accessible at https://ngdc.cncb.ac.cn/gsa‐human. Any additional information required to reanalyze the data reported in this work paper is available from the lead contact upon request.
