# Supplementary material for: Multi‐Omics Data Reveal Estrogen‐Driven Dysregulation and Stromal‐Epithelial Signaling Alterations in Endometrial Polyps
Source: FASEB J. 2026 Mar 5;40(5):e71645. doi: 10.1096/fj.202504234R (PMC12963523; doi:10.1096/fj.202504234R)
Supplement: Supplementary file 1 — Data S1: fsb271645‐sup‐0001‐Supinfo.docx. [file FSB2-40-e71645-s001.docx]

Multi-omics data reveal estrogen-driven dysregulation and stromal-epithelial signaling alterations in endometrial polyps

Tingwei Chen^1,2,3#^, Bo Zhang^1,2,3#^, Zhengli Zhou^4#^, Naixue Yang^1,2#^, Ting Liu^1,2^, Huimei Zhang^1,2,3^, Yu Yin^1,2^, Xiaomei Wu^4^, Xiaozhuo Li^1,2^,Tao Yu^4^ ,Xiaodie Wang^4^, Tianqing Li^1,2,3^* ,E Dong^1,2^*

1. State Key Laboratory of Primate Biomedical Research; Institute of Primate Translational Medicine, Kunming University of Science and Technology, Kunming 650500, China.

2. Yunnan Key Laboratory of Primate Biomedical Research, Kunming 650500, China.

3. Southwest United Graduate School, Kunming, 650092, China

4. The First people’ Hospital of Yunnan Province; the Affiliated Hospital of Kunming University of Science and Technology, Kunming, 650031, China.

These authors contributed equally: Tingwei Chen, Bo Zhang, Zhengli Zhou, Naixue Yang.

Corresponding author：Tianqing Li, [litq@lpbr.cn;](mailto:litq@lpbr.cn;) E Dong, [donge@lpbr.cn](mailto:donge@lpbr.cn).

**This PDF file includes:**

**Supplementary table 1**

**Supplementary Figure 1 to 9**

**Supplementary Table 1:Endometrium tissue and sampling.**

| Case # | Block | Tissue sampling | # of cells | Fraction reads in cell | Median genes per cell | Sex | Age | Period |
| --- | --- | --- | --- | --- | --- | --- | --- | --- |
| Polyp1  20210317-D2 | Polyp1 | Tissue adjacent to polyps | 8,447 | 73.0% | 1,309 | F | 27 | secretory |
| Polyp2  20210722-4 | Polyp3 | Polyps tissue | 18,950 | 79.1% | 2,008 | F | 39 | proliferaion |
| Polyp3  20210722-5 | Polyp4 | Polyps tissue | 12,463 | 69.5% | 1,578 | F | 34 | proliferaion |
| Control1  20200513-B1 | CTRL1 | Normal endometrium | 18,211 | 92.0% | 2,568 | F | 25 | proliferaion |
| Polyp4  20200513-B2 | Polyp2 | Tissue adjacent to polyps | 20,773 | 89.9% | 1,782 | F | 33 | proliferaion |
| Control2  20200520-A9 | CTRL2 | Normal endometrium | 13,857 | 79.4% | 2,315 | F | 30 | proliferaion |

*Abbreviations: IUA=intrauterine adhesion; M=male; F=female*


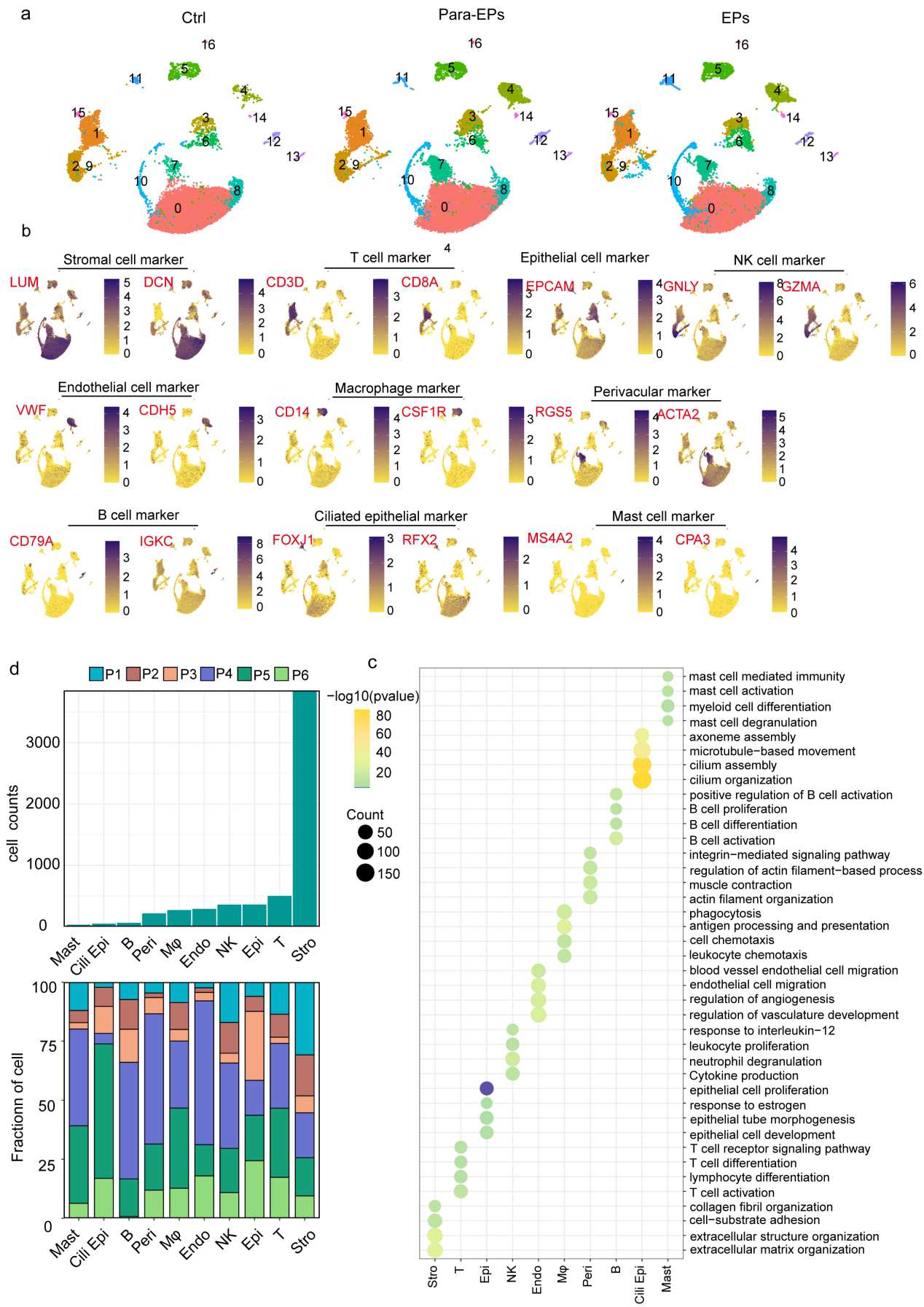


**Fig. S1 Single-cell transcriptomic landscape of human endometrium from control, para-EPs, and EPs tissues. (a)** UMAP plots showing cell clustering results of integrated single-cell transcriptomic profiles from control, para-polyp, and polyp groups. Each dot represents a single cell, and colors denote identified cell types. **(b)** Feature plots showing the expression of canonical marker genes for stromal cells (LUM, DCN), T cells (CD3D, CD8A), epithelial cells (EPCAM), NK cells (GNLY, GZMA), endothelial cells (VWF, CDH5), macrophages (CD14, CSF1R), perivascular cells (RGS5, ACTA2), B cells (CD79A, IGKC), ciliated epithelial cells (FOXJ1, RFX2), and mast cells (MS4A2, CPA3). **(c)** Bar plots showing the relative composition of cell types (bottom) and the total cell counts (top) across six individual patient samples (P1-P6). **(d)** GO enrichment analysis of differentially expressed genes across major cell types, highlighting biological processes enriched in each population. Circle size indicates the number of genes associated with each term; color denotes statistical significance (−log_10_p-value).

**Fig. S2 Increased PGR expression and epithelial proliferation in endometrial polyps.**
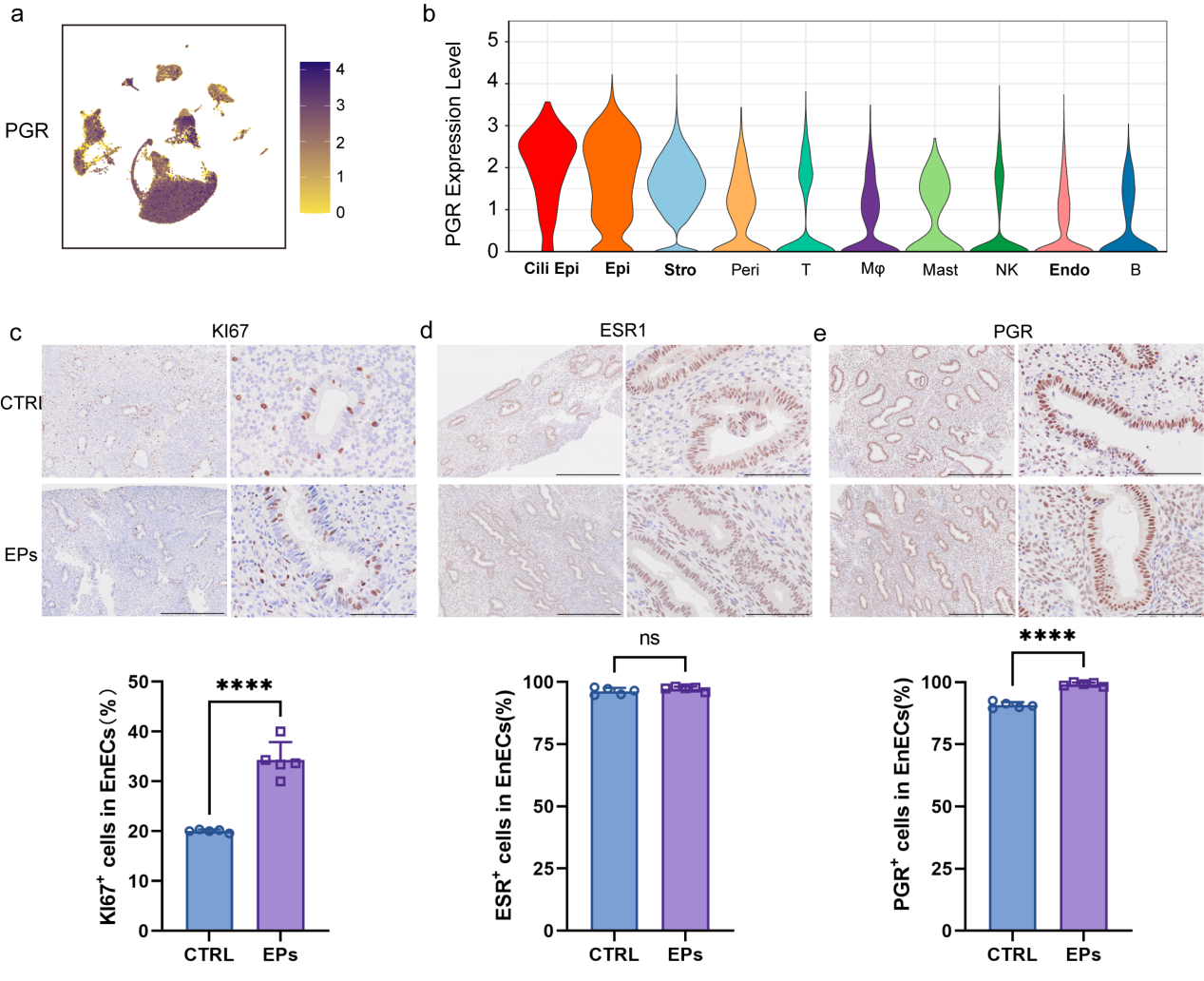


**(a)** UMAP plot of PGR expression in normal and EPs cells. The color scale represents normalized gene expression. **(b)** Expression levels of PGR across all ten cell types. **(c-e)** Representative immunohistochemical staining and quantification of Ki67-, ESR1- and PGR-positive epithelial cells in control endometrium (CTRL) and endometrial polyps (EPs). Data are presented as mean ± SEM. Statistical significance was determined by unpaired two-tailed Student’s t-test. ****P < 0.0001; ns, not significant.Scale bar:100μm.


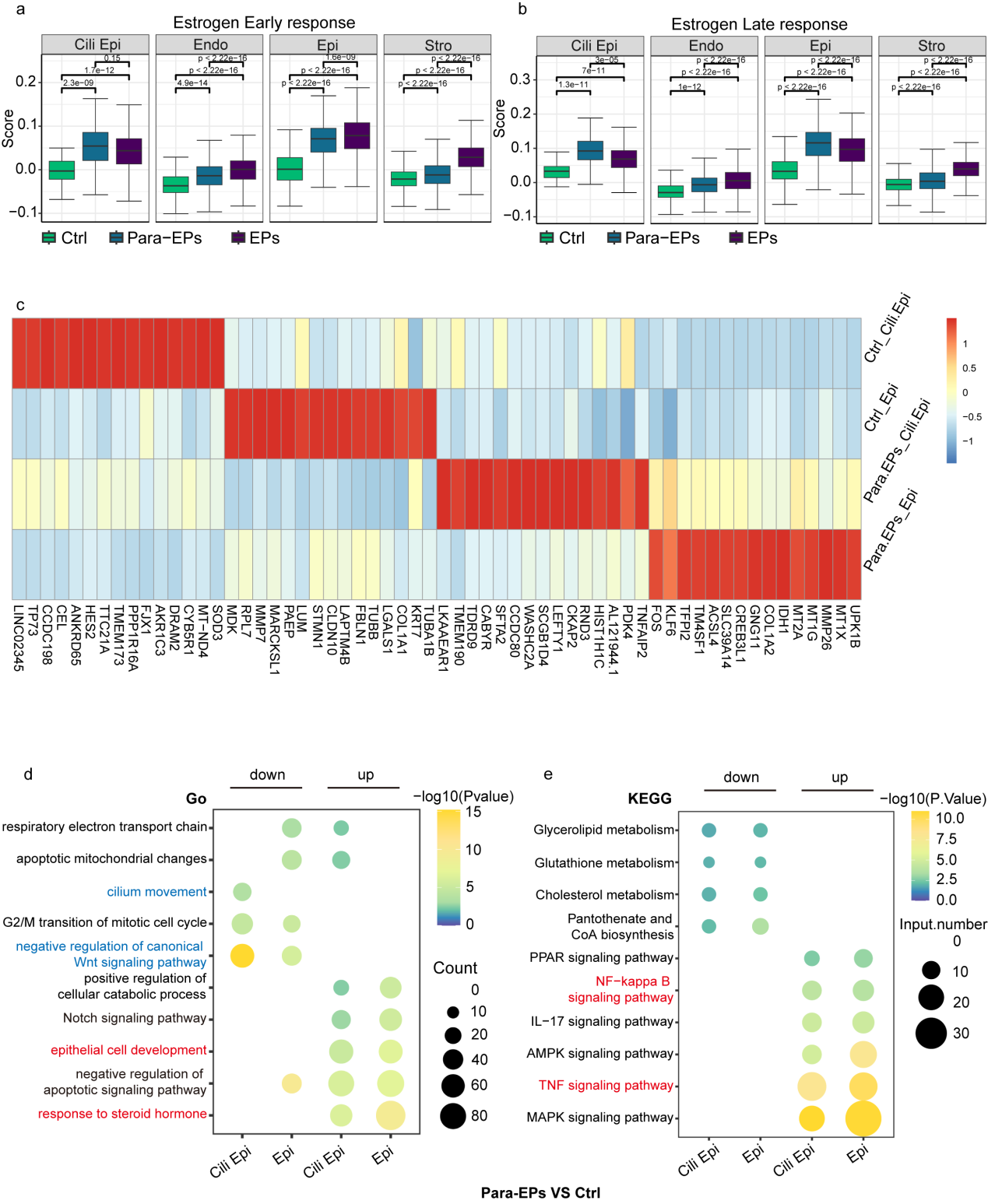


**Fig. S3 Enhanced estrogen responsiveness and transcriptional reprogramming in epithelial cells of endometrial polyps. (a-b)** Boxplots showing estrogen early response **(a)** and late response **(b)** scores across four major structural cell types-ciliated epithelial cells (Cili Epi), endothelial cells (Endo), epithelial cells (Epi), and stromal cells (Stro)-in control (Ctrl), para-polyp (Para-EPs), and polyp (EPs) groups. (two-sided Wilcoxon rank-sum test). **(c)** Heatmap showing the top 15 differentially expressed genes (DEGs) in epithelial cell subsets across Ctrl, Para-EPs, and EPs groups. Gene expression is scaled across all groups (red: high; blue: low). **(d–e)** Bubble plots of Gene Ontology **(d)** and KEGG pathway **(e)** enrichment analyses of DEGs between Para-EPs and Ctrl in epithelial subtypes. Downregulated (left) and upregulated (right) pathways are shown for Cili Epi, Epi. Dot size reflects gene count, and color indicates -log_10_ P-value.


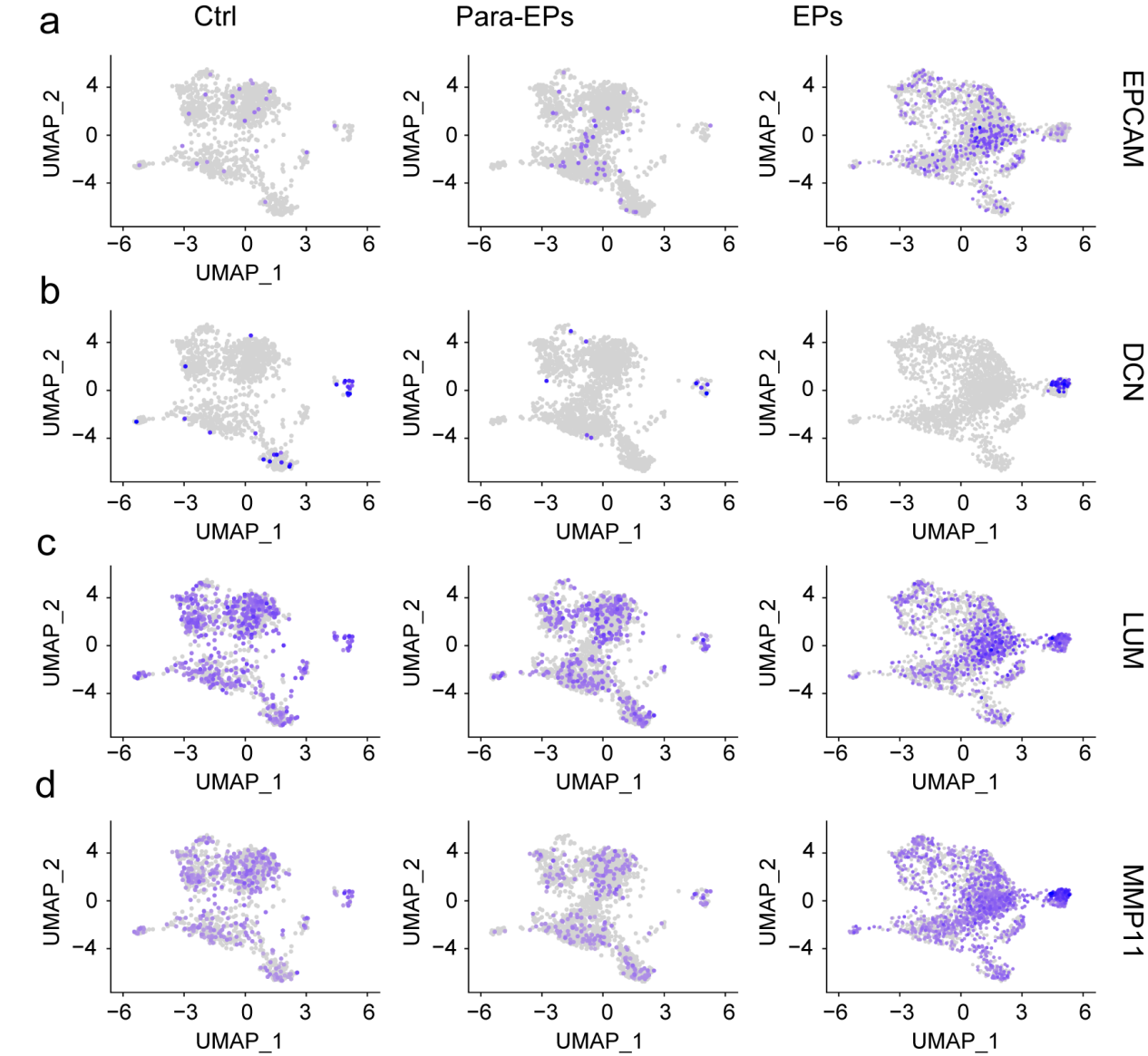
**Fig. S4** UMAP visualization of cell-type-specific marker gene expression across ctrl, para-EPs, and EPs endometrial samples.**(a-d)** UMAP plots showing the expression of representative marker genes across the ctrl, para-EPs, and EPs groups. **(a)** EPCAM, a canonical epithelial cell marker; **(b)** DCN, a fibroblast/stromal marker; **(c)** LUM, an extracellular matrix-associated stromal marker; **(d)** MMP11, a matrix metalloproteinase associated with tissue remodeling


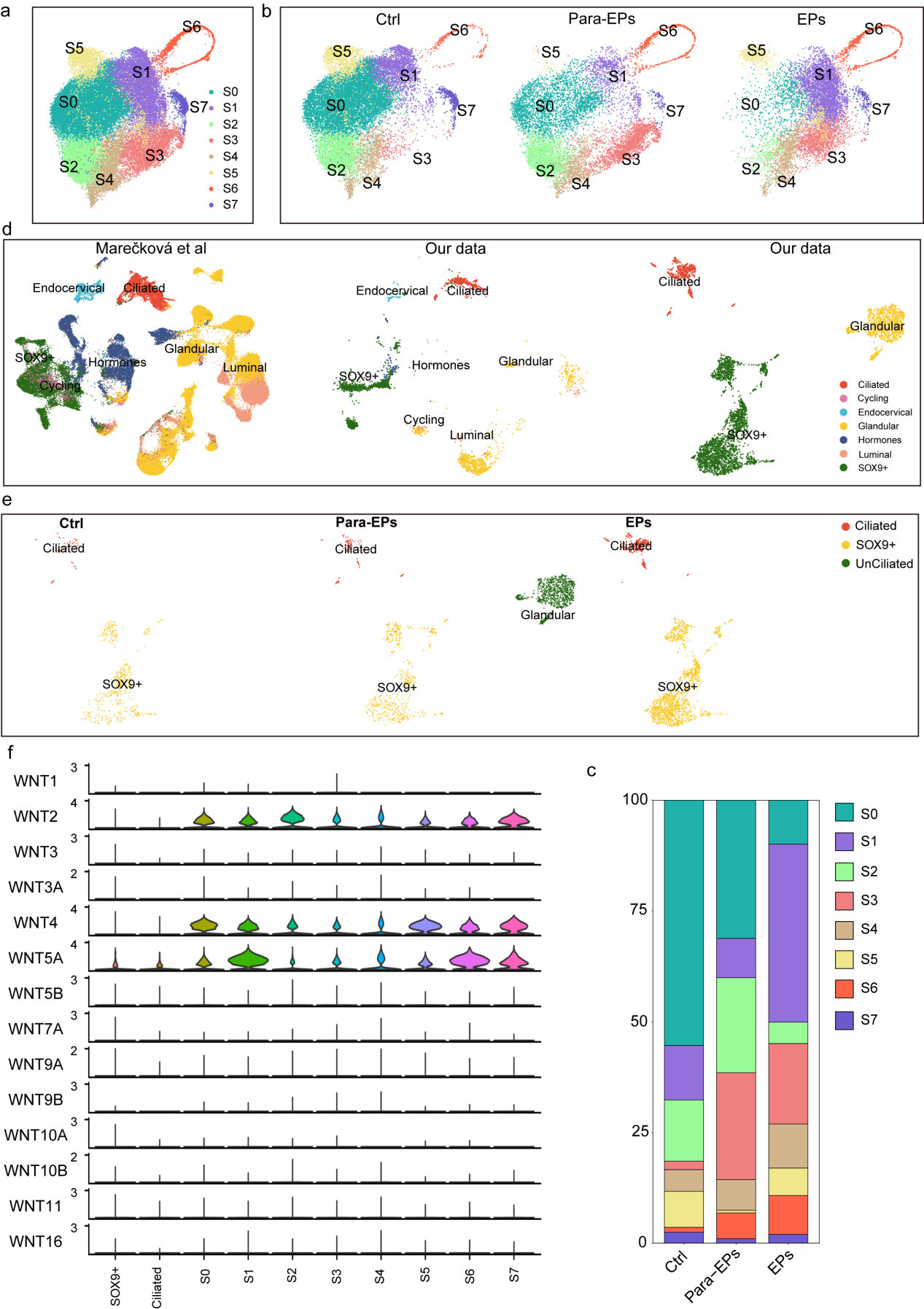


**Fig. S5 The heterogeneity of stromal cells and epithelial cells, as well as the expression of WNT ligands in both cell types. (a)** UMAP projection of stromal cells across all samples, revealing eight transcriptionally distinct subpopulations (S0-S7). **(b)** UMAP plot showing distribution of the stromal subpopulations (S0-S7) across Ctrl, Para-EPs, and EPs groups. **(c)** Proportional bar plots showing the relative abundance of each stromal subset across the three groups. **(d)** Reference-based annotation of epithelial cells. UMAP plots show label transfer results using Marečková et al.’s Human Endometrial Cell Atlas as reference (left), with corresponding annotations mapped onto our dataset (middle). Based on this, epithelial cells were classified into SOX9⁺, ciliated, and glandular subtypes (right). **(e)** UMAP visualization of epithelial subtypes (SOX9⁺, ciliated, and unciliated) across the Ctrl, Para-EPs, and EPs groups. **(f)** Violin plots showing expression patterns of WNT ligand genes across stromal subsets (S0-S7) and epithelial subtypes (SOX9⁺ and ciliated).


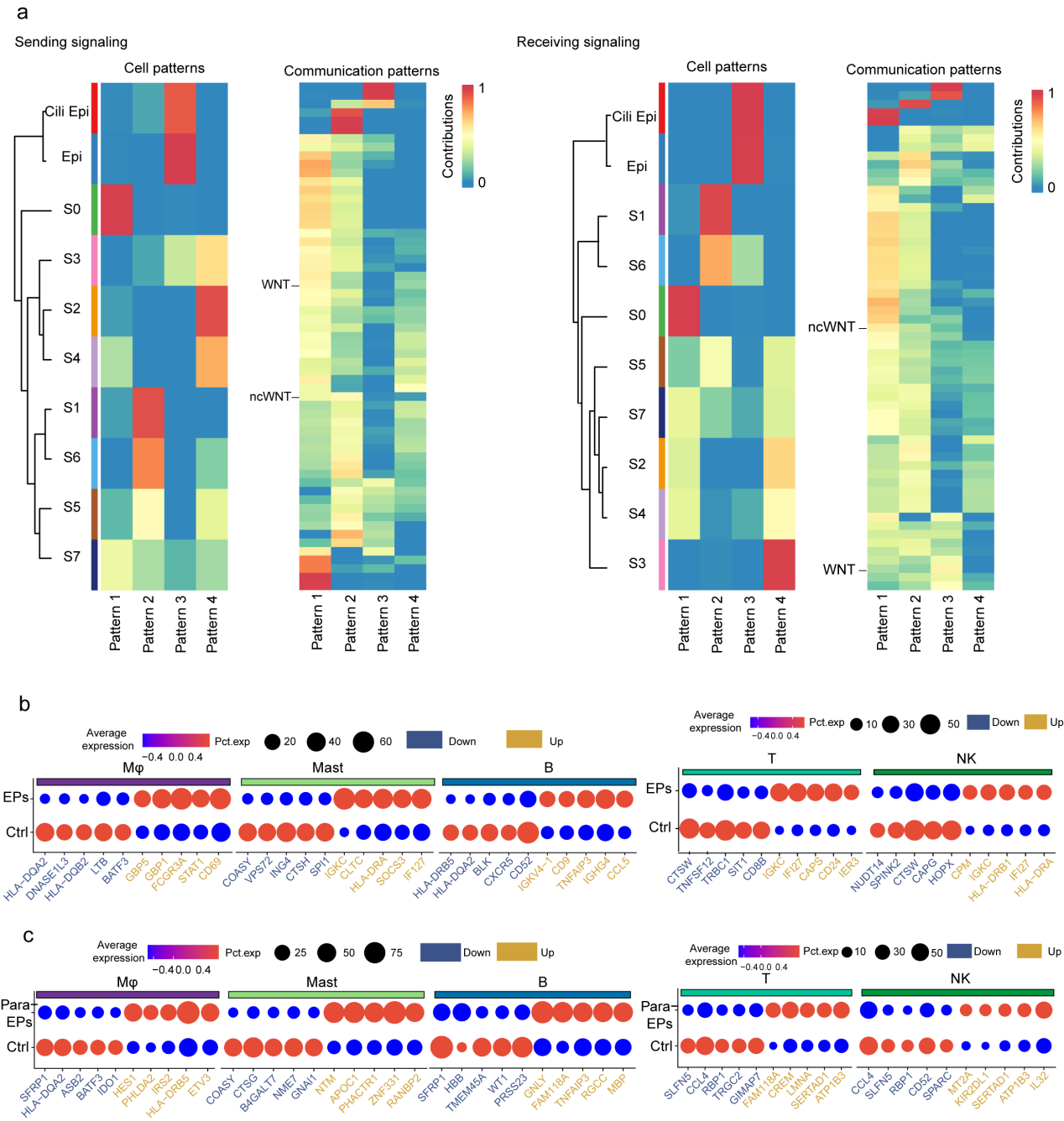
**Fig. S6 Communication between epithelial and stromal subsets.** **(a)** Heatmaps showing CellChat-inferred outgoing (left) and incoming (right) signaling patterns across stromal and epithelial subsets. Columns represent cell types, and rows denote signaling pathway patterns. Communication strength (contribution scores) is represented by color scale (blue to red), with hierarchical clustering revealing group-specific signaling architecture. **(b-c)** Dotplots showing the five most upregulated or downregulated DEGs (control versusEPs **b,** control versus para-EPs, c) in each immune cell types.


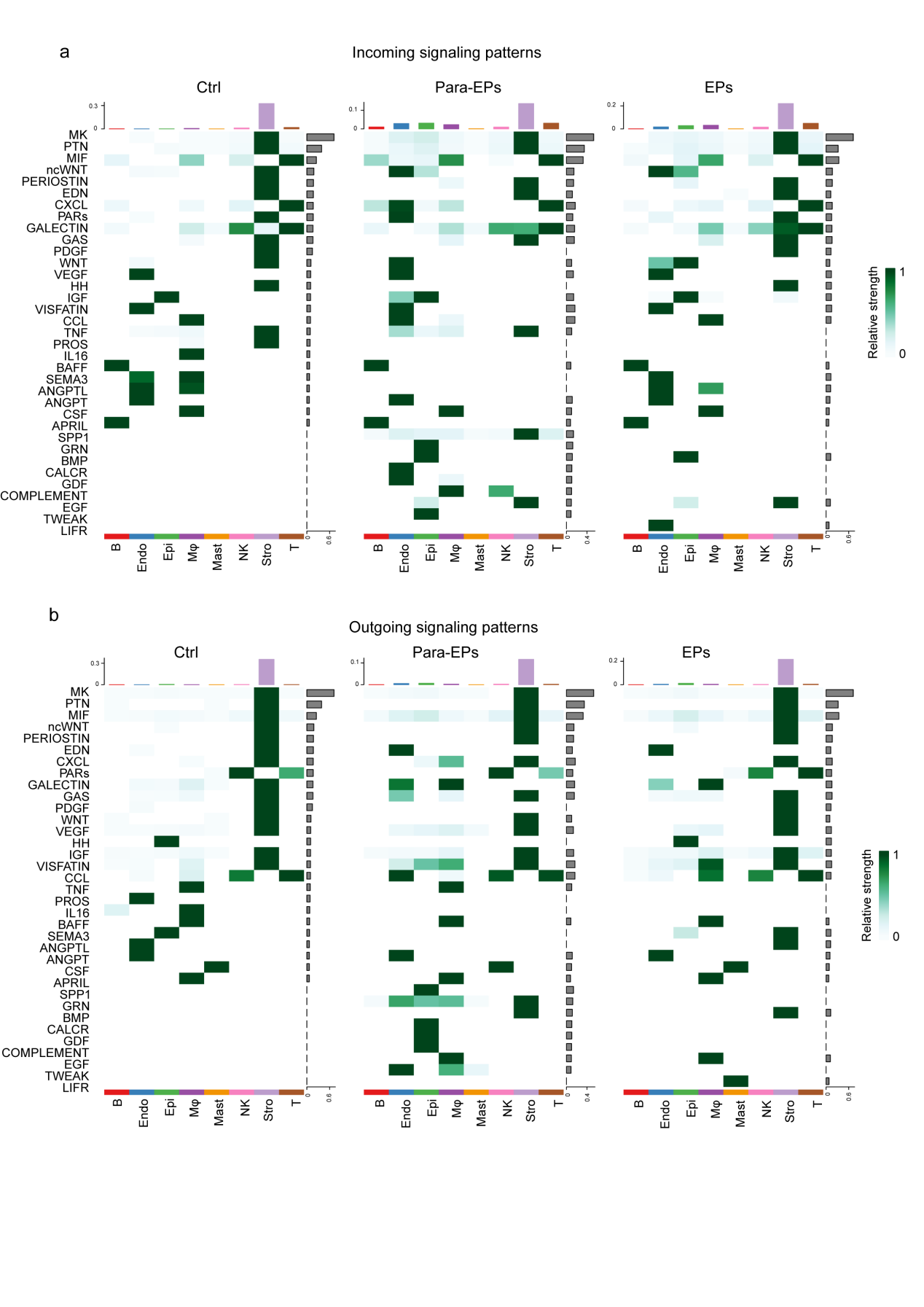


**Fig. S7 Remodeled intercellular signaling landscape in endometrial polyps .(a)** Heatmaps showing relative strength of incoming signaling patterns for each cell type across Ctrl, Para-EPs, and EPs groups, based on CellChat analysis. Each row represents a signaling pathway, and each column represents a major cell type. **(b)** Heatmaps showing relative strength of outgoing signaling patterns for each cell type across the same groups.


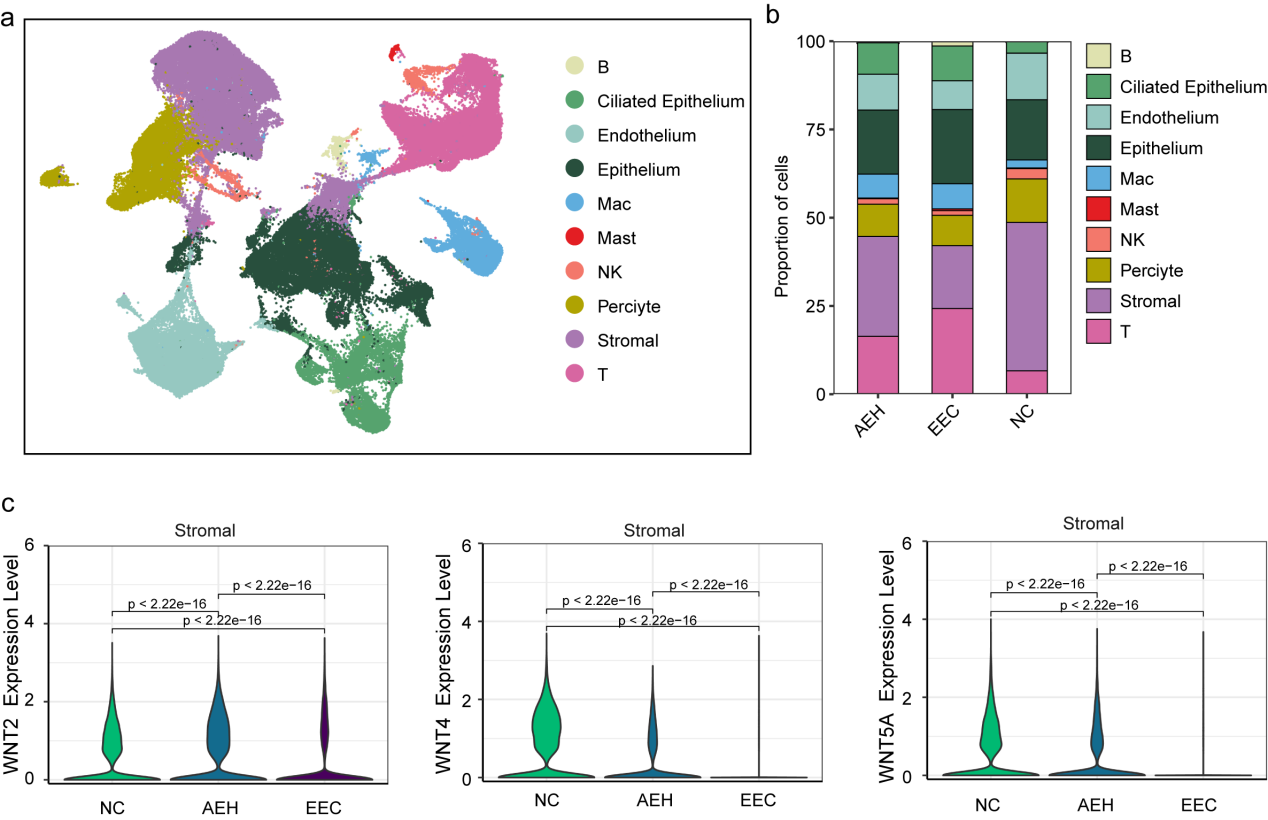


**Fig. S8 Single-cell atlas of endometrial cancer from Ren et al. (a)** The UMAP plot illustrates 10 cell types in endometrial cancer. **(b)** The column chart shows the proportions of each cell in the three groups. **(c)** Expression levels of Wnt2, Wnt4, and Wnt5a in stromal cells from the three groups (NC, AEH, EEC).

**
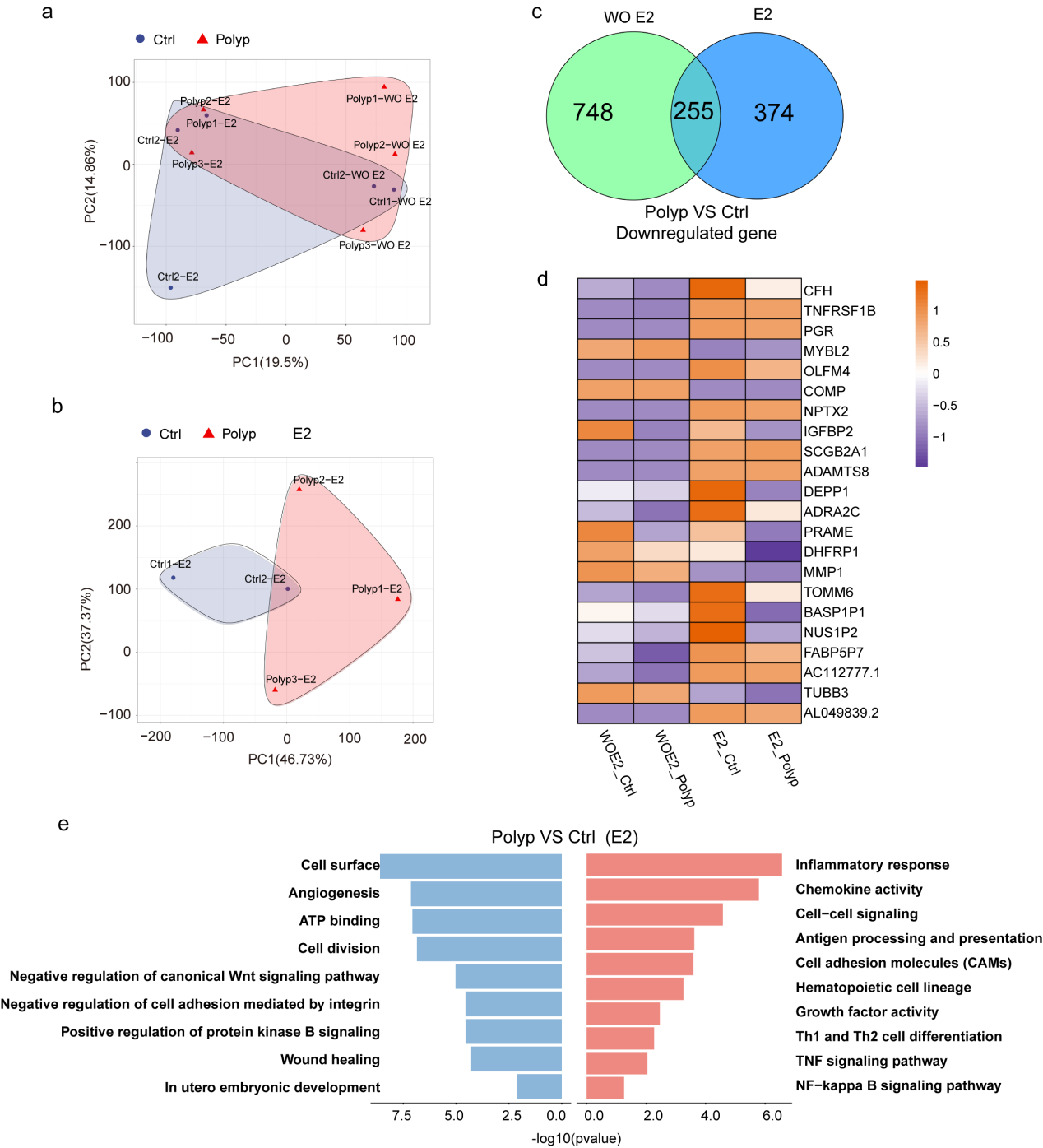
**

**Fig. S9 Transcriptomic alterations in epithelial cells. (a,b)** Principal component analysis (PCA) for bulk-RNA sequencing data of tissue samples from polyp and control groups under E2 treatment or untreated conditions. **(c)** The Venn diagram depicts genes downregulated in polyps relative to controls without E2 treatment, and the overlapping genes downregulated under E2 treatment. **(d)** Heatmap displaying the top 22 genes down-regulated in polyps under both E2-treated and untreated conditions relative to controls. **(e)** KEGG and GO enrichment analyses of differentially expressed genes between polyps and controls under E2 treatment; blue indicates down-regulation, red indicates up-regulation.
